# Supplementary material for: Molecular and functional heterogeneity in dorsal and ventral oligodendrocyte progenitor cells of the mouse forebrain in response to DNA damage
Source: Nat Commun. 2022 Apr 28;13:2331. doi: 10.1038/s41467-022-30010-6 (PMC9051058; doi:10.1038/s41467-022-30010-6)
Supplement: Supplementary file 1 — Supplementary Information [file 41467_2022_30010_MOESM1_ESM.pdf]

Supplementary Information

Molecular and functional heterogeneity in dorsal and ventral oligodendrocyte progenitor cells of the mouse forebrain in response to DNA damage

Enrica Boda, Martina Lorenzati, Roberta Parolisi, Brian Harding, Gianmarco Pallavicini, Luca Bonfanti, Amanda Moccia, Stephanie Bielas, Ferdinando Di Cunto, Annalisa Buffo

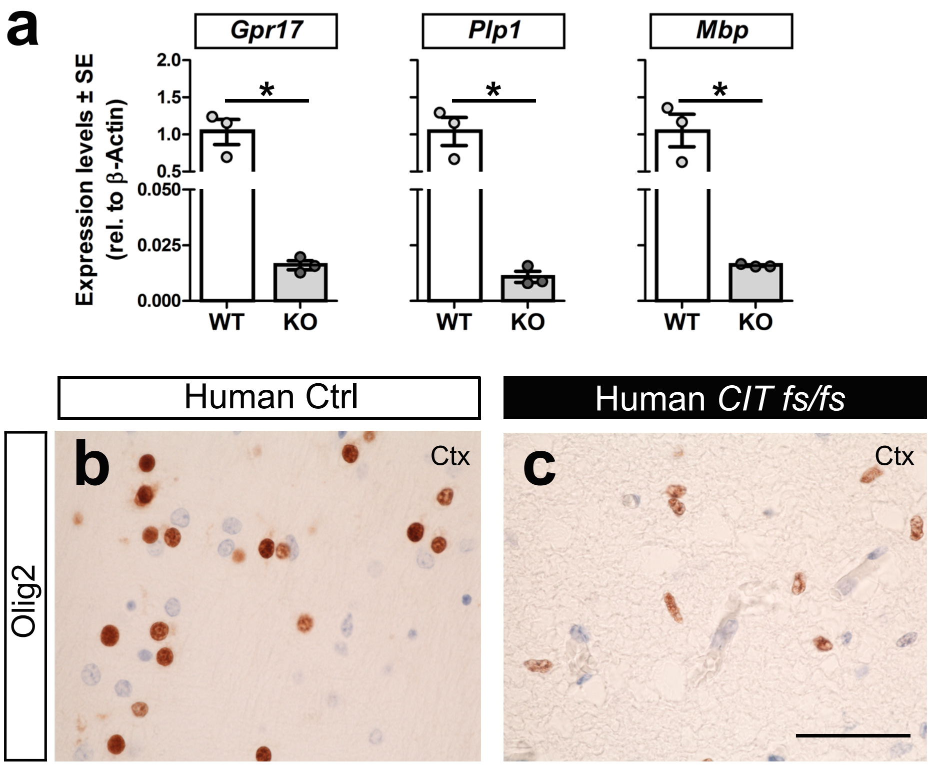

Supplementary Figure 1. Oligodendroglial defects in *Cit-k KO* mouse and *CIT-K fs/fs* human brain

Supplementary Figure 1. Oligodendroglial defects in *Cit-k KO* mouse and *CIT-K fs/fs* human brain

(a) Reduced mRNA expression levels of Gpr17 (marker of premyelinating oligodendrocytes), Plp1 and Mbp (markers of mature oligodendrocytes) in P14 *Cit-k KO* vs. WT mouse forebrain (n=3 each, *, P = 0.05, one-tailed Mann–Whitney U-test). Data are mean ± SEM**. (b,c)** Representative images of anti-Olig2 immunostaining in human newborn (P1) Ctrl **(b)** and *CIT-K fs/fs* **(c)** cortices. Scale bar: 50 µm. Source data are provided as a Source Data file.

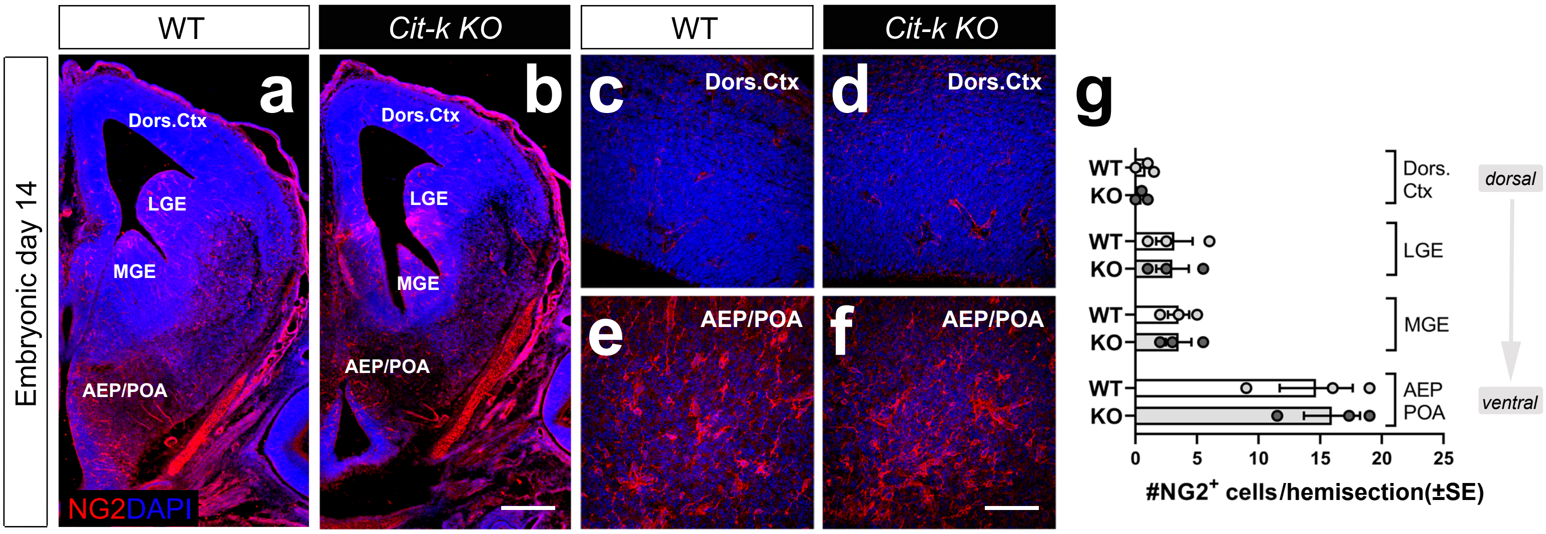

Supplementary Figure 2. Distribution and abundance of NG2+ cells do not differ in E14 *Cit-k KO* vs. WT mouse forebrain

Supplementary Figure 2. Distribution and abundance of NG2$^+$ cells do not differ in E14 *Cit-k KO* vs. WT mouse forebrain

(a,b) Low magnification representative image of E14 WT **(a)** and Cit-k KO **(b)** forebrain immunostained with an anti-NG2 antibody (red). **(c-f)** Representative images of the distribution and abudance of NG2-expressing (red) cells in the dorsal cortex **(c,d)** and AEP/POA **(e,f)** of E14 WT **(c,e)** and *Cit-k KO* (**d,f**) mice at embryonic day 14 (E14). DAPI (blue) counterstains cell nuclei. (**g**) Quantification of the absolute numbers/hemisection of NG2$^+$ cells in distinct forebrain regions at E14. Two-tailed Mann-Whitney test reveals no significant differences between WT and *KO* in all regions (n=3 each, n.s.). Data are mean ± SEM. Scale bars: 250 µm in **a,b**; 50 µm in **c-f**. Abbreviations: AEP/POA, anterior entopeduncular/preoptica area; Dors. Ctx., dorsal cortex; LGE, lateral ganglionic eminence; MGE, medial ganglionic eminence. Source data are provided as a Source Data file.

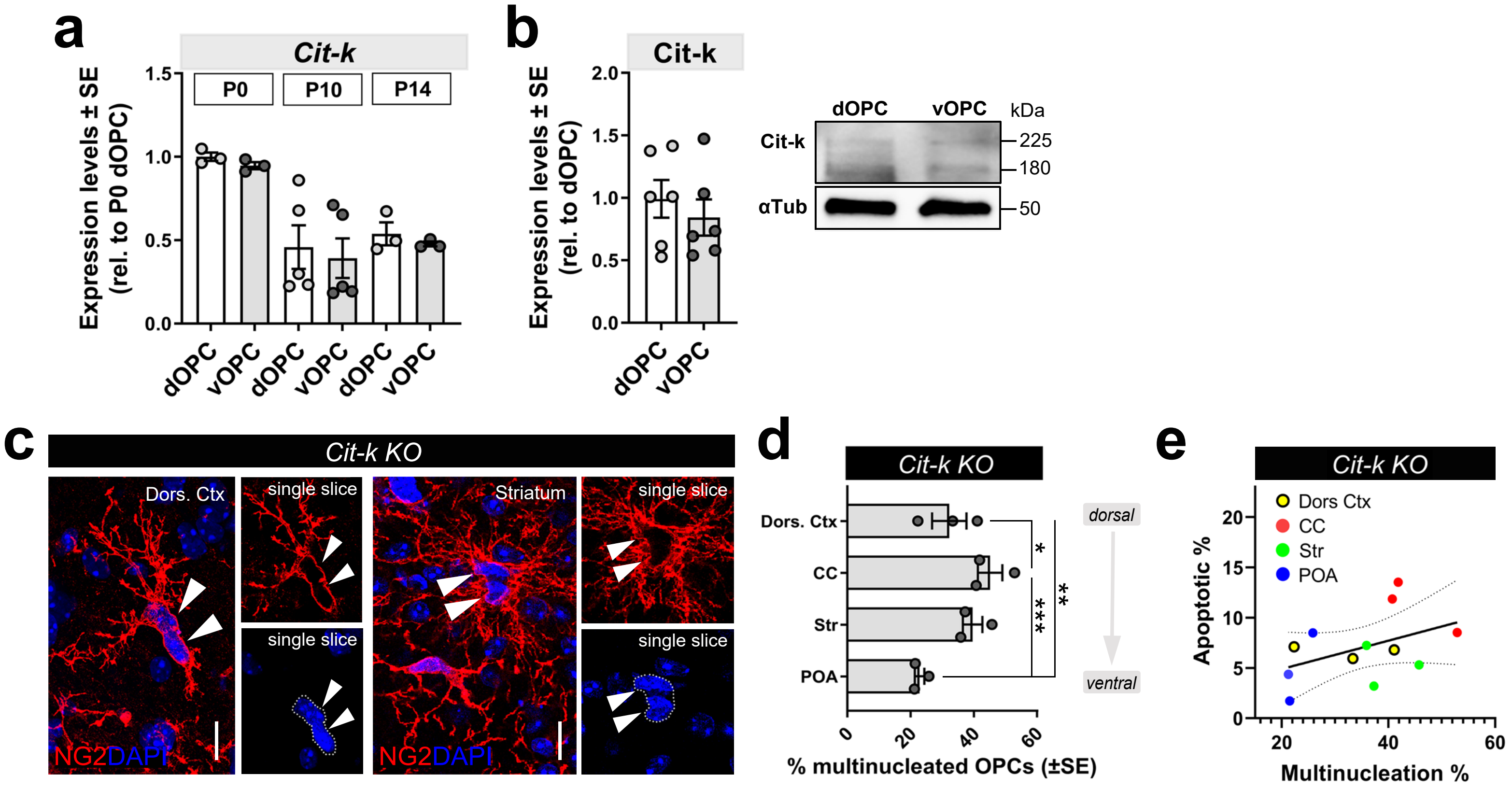

Supplementary Figure 3. CIT-K is ubiquitously expressed in postnatal mouse forebrain OPCs

Supplementary Figure 3. CIT-K is ubiquitously expressed in postnatal mouse forebrain OPCs

(a) *Cit-k* mRNA expression levels in OPCs MACSorted from P0 (n=3 each), P10 (n=5 each) and P14 (n=3 each) WT mouse forebrain. dOPCs were isolated from the dorsal cortex. vOPCs were isolated from the ventral forebrain. Differences between groups were assessed by Two-way Anova followed by Bonferroni's Multiple Comparison Test (Region effect: n.s. $F(1,16)=0.460$; Time effect: $P=0.0003$ $F(2,16)=14.503$; Region x Time: n.s. $F(2,16)=0.002$) **(b)** Western Blot quantification of CIT-K protein expression reveals no difference in WT P8 dOPCs vs vOPCs (n=6 each, Two-tailed paired t-test, n.s., $t(5)=0.516$). Note that the anti-CIT antibody recognizes both CIT-K (225 kDa) and CIT-N (180 kDa) isoforms. **(c)** Representative images of binucleated $NG2^+$ (red) OPCs in P3 *Cit-k KO* dorsal cortex and striatum. DAPI (blue) counterstains cell nuclei. White arrowheads indicate cells with 2 nuclei. Scale bars: 10 µm. **(d)** Quantification of the percentage of multinucleated cells in distinct regions along the dorso-ventral axis of P10 Cit-k KO mouse brain (n=3; Chi-square test, $P<0.0001$, $\chi^2(3)=45.63$). **(e)** Linear regression analysis of the percentage of apoptotic OPCs vs percentage of multinucleated OPCs in distinct P10 *Cit-k KO* brain regions (n=3, $P=0.1557$, the slope is not significantly different to 0; $y=0.141x+2.067$, $R^2=0.1981$, $Sy.x=3.169$). Data in **a,b,d** are mean ± SEM. Abbreviations: CC, corpus callosum; Dors. Ctx, dorsal cortex; dOPC, dorsal oligodendrocyte progenitor cells; POA, preoptic area; Str, striatum; vOPC, ventral oligodendrocyte progenitor cells. *, $P<0.05$; **, $P<0.01$; ***, $P<0.001$. Source data are provided as a Source Data file.

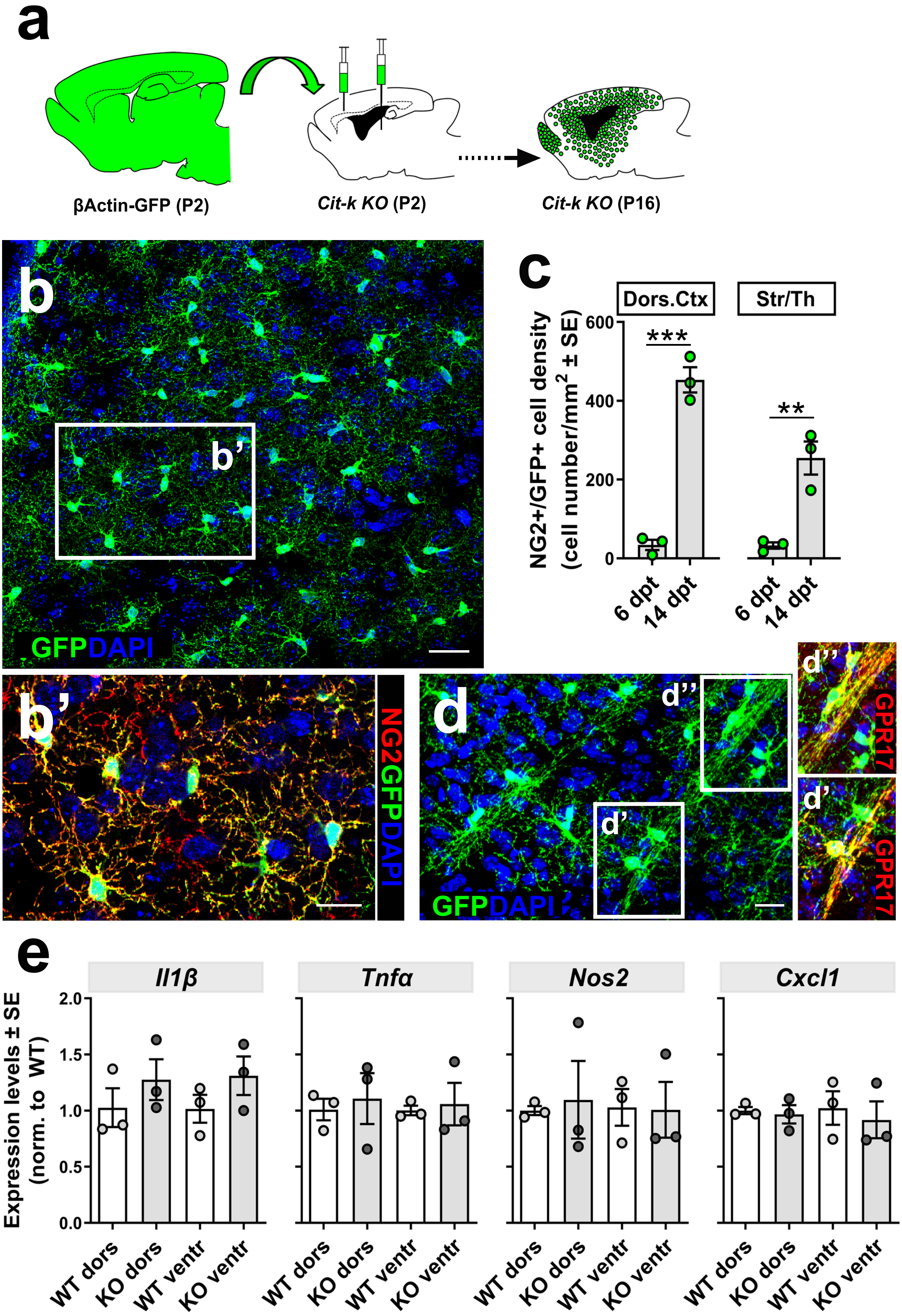

Supplementary Figure 4. Grafted WT OPCs survive and expand in *Cit-k KO* mouse forebrain

Supplementary Figure 4. Grafted WT OPCs survive and expand in *Cit-k KO* mouse forebrain

(a) Representative scheme of the grafting approach: cells from P2 βActin-GFP mouse dorsal cortex were transplanted in the deep cortex/white matter and striatum of P2 *Cit-k KO* mice. Transplanted cells colonized the entire forebrain. **(b)** The large majority of grafted (green) cells were $NG2^+$ (red in **b'**, single slice) OPCs. **(c)** Quantification of the density of $NG2^+$ transplanted cells in the dorsal cortex and striatum/thalamus of *Cit-k KO* mice at distinct days post-transplantation reveals a progressive expansion (n=3/each; Two-tailed Unpaired t-test, Dors. Ctx: P=0.0003, t(4)=12.15; Str/Thal: P=0.0064, t(4)=5.22), indicating that grafted cells neither die and suggesting they neither acquire senescent features. **(d)** Some premyelinating $GPR17^+$ (red in **d',d''**) oligodendrocytes were also found among transplanted (green) cells in the subcortical white matter and striatum. **(e)** qRT-PCR analysis of the mRNAs coding for proinflammatory factors reveals no upregulation in *Cit-k KO* dorsal or ventral tissue compared to WT (n=3 each, Kruskal-Wallis Test, n.s.). Data are mean ± SEM. Scale bars: 20 µm. Abbreviations: Dors. Ctx., dorsal cortex; DPT, days post-transplantation; GFP, green fluorescent protein; P, postnatal day; WT, wild-type. **, P<0.01; ***, P<0.001. Source data are provided as a Source Data file.

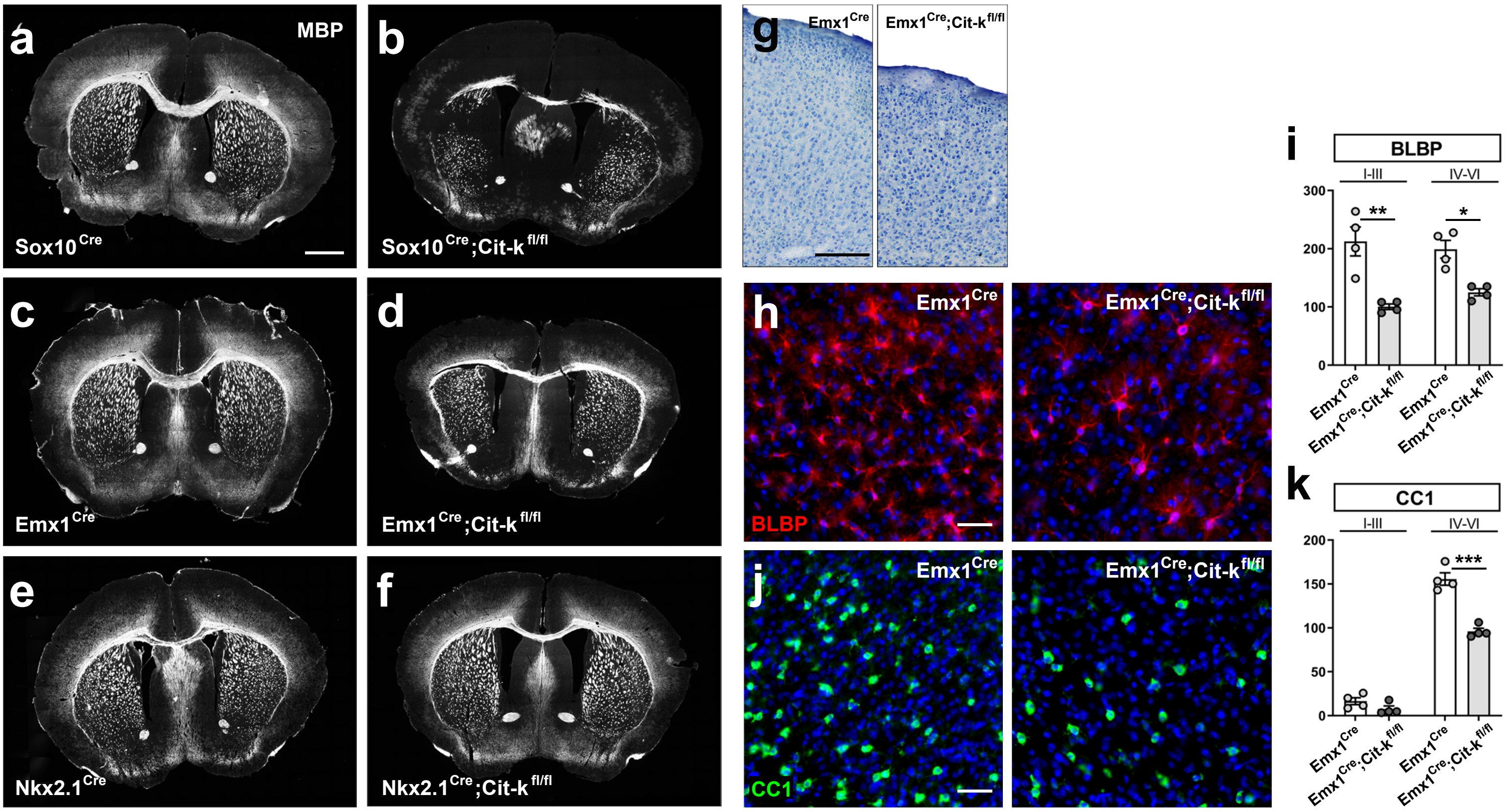

Supplementary Figure 5. MBP expression pattern in conditional Cit-k$^{fl/fl}$ mouse lines and phenotype of Emx1^{Cre};Cit-k$^{fl/fl}$ mouse cortex

Supplementary Figure 5. MBP expression pattern in conditional Cit-k$^{fl/fl}$ mouse lines and phenotype of Emx1^{Cre};Cit-k$^{fl/fl}$ mouse cortex

(a-f) Representative images of MBP staining (white) in P14 Sox10^{Cre} (Ctrl, **a**) *vs.* Sox10^{Cre};Cit-k$^{fl/fl}$ **(b)**, Emx1^{Cre} (Ctrl, **c**) *vs.* Emx1^{Cre};Cit-k$^{fl/fl}$ **(d)**, Nkx2.1^{Cre} (Ctrl, **e**) *vs.* Nkx2.1^{Cre};Cit-k$^{fl/fl}$ **(f)**. **(g)** Representative images of Nissl staining (blue) in P14 Emx1^{Cre} *vs.* Emx1^{Cre};Cit-k$^{fl/fl}$ dorsal cortex. Representative images **(h)** and quantification **(i)** of BLBP^{+} (red) astrocytes in layers I-III and IV-VI of P14 Emx1^{Cre} *vs.* Emx1^{Cre};Cit-k$^{fl/fl}$ dorsal cortex (n=4 mice/group; Two-way Anova followed by Bonferroni's Multiple Comparison Test, Genotype effect: $P<0.0001$, $F(1,12) = 38.15$; Region effect: n.s., $F(1,12) = 0.1404$; Genotype x Region: n.s., $F(1,12) = 1.612$). Representative images **(j)** and quantification **(k)** of CC1^{+} (green) mature oligodendrocytes in layers I-III and IV-VI of P14 Emx1^{Cre} *vs.* Emx1^{Cre};Cit-k$^{fl/fl}$ dorsal cortex (n=4 mice/group; Two-way Anova followed by Bonferroni's Multiple Comparison Test, Genotype effect: $P<0.0001$, $F(1,12) = 52.36$; Region effect: $P<0.0001$, $F(1,12) = 580.5$; Genotype x Region: $P=0.0002$, $F(1,12) = 28.76$). Data are mean ± SEM. ***, $P<0.001$; **, $P<0.01$, *, $P<0.05$. Scale bars: 500 μm in **a-f**; 200 μm in **g**; 50 μm in **h,j**. Source data are provided as a Source Data file.

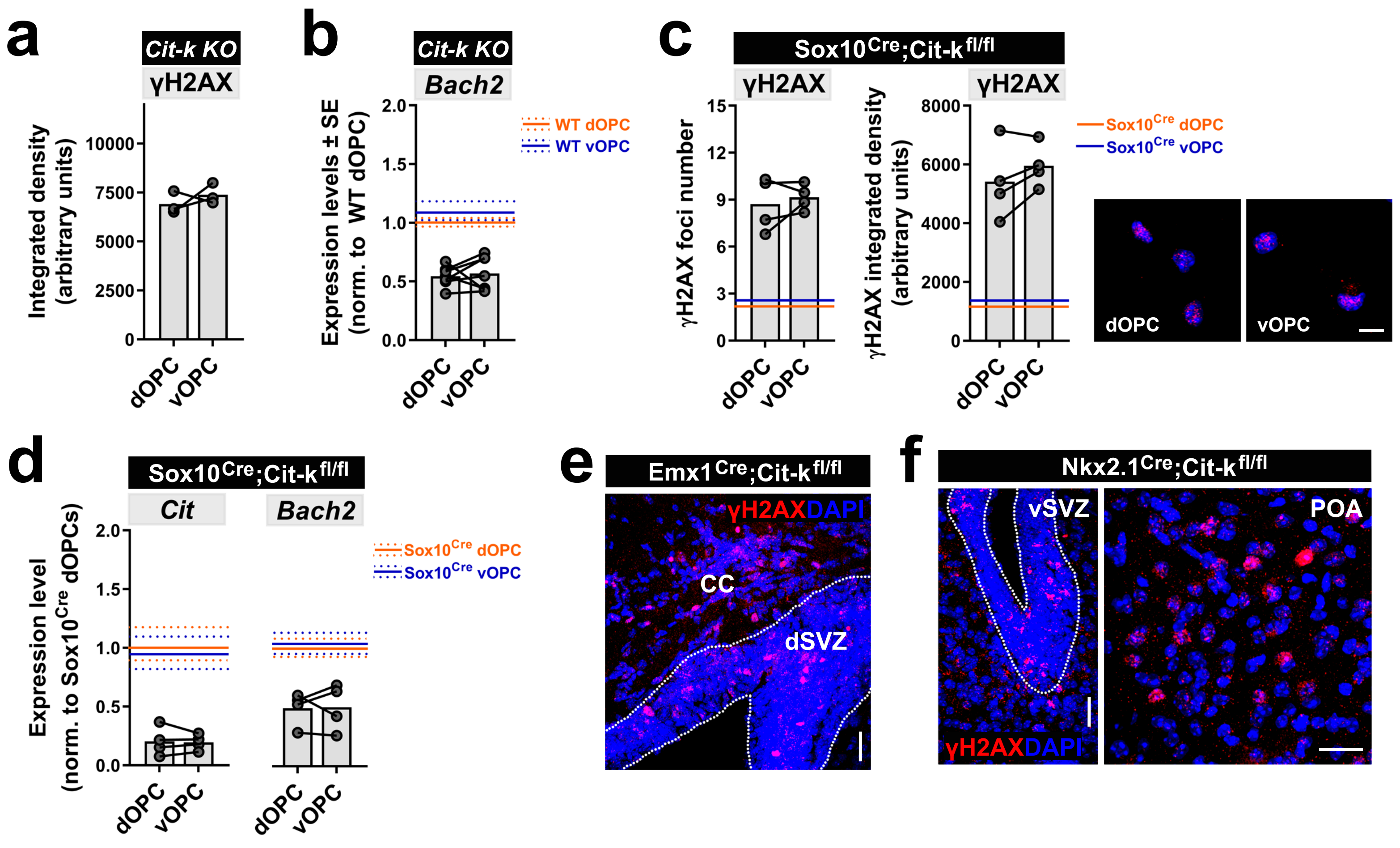

Supplementary Figure 6. Equivalent accumulation of DNA damage in dorsal and ventral OPCs of P10 *Cit-k KO* and conditional Cit-k$^{fl/fl}$ mouse brain

Supplementary Figure 6. Equivalent accumulation of DNA damage in dorsal and ventral OPCs of P10 *Cit-k KO* and conditional Cit-k$^{fl/fl}$ mouse brain

(a) Quantification of the nuclear γH2AX integrated density (i.e. mean intensity multiplied by the area) in dOPCs and vOPCs MACSorted from P10 *Cit-k KO* mice (n=3 each, two-tailed Mann–Whitney U-test, n.s.). **(b)** *Bach2* mRNA expression levels in dOPCs and vOPCs MACSorted from P10 WT and *Cit-k KO* mice (n=7 each; Two-way Anova, Genotype: P<0.0001, F(1,25)=112.1; Region: n.s., F(1,25)=1.668; Genotype x Region: n.s., F(1,25)=0.648). Orange and blue lines represent mean (solid line) ± SE (dotted lines) of WT dOPCs and WT vOPCs, respectively. **(c)** Quantification of γH2AX^{+} foci and γH2AX integrated density reveals no differences between Sox10^{Cre};Cit-k$^{fl/fl}$ dOPCs (n=4 mice) and vOPCs (n=4 mice; two-tailed Mann–Whitney U-test, n.s.). Orange and blue lines represent mean (solid line) ± SE (dotted lines) of Sox10^{Cre} (Ctrl) dOPCs and vOPCs, respectively. Representative images of anti-γH2AX immunolabeling in Sox10^{Cre};Cit-k$^{fl/fl}$ dOPCs and vOPCs. **(d)** *Cit-k* and *Bach2* mRNA expression levels in dOPCs and vOPCs MACSorted from P10 Sox10^{Cre} (Ctrl, n=4) vs. Sox10^{Cre};Cit-k$^{fl/fl}$ (n=4) mouse forebrain. Orange and blue lines represent mean (solid line) ± SE (dotted lines) of Sox10^{Cre} (Ctrl) dOPCs and vOPCs, respectively. Differences between groups were assessed by Two-way Anova followed by Bonferroni's Multiple Comparison Test (*Cit-k* Region effect: n.s. F(1,12)=0.146; Genotype effect: P<0.0001 F(1,12)=58.0; Region x Genotype: n.s. F(1,12)=0.099; *Bach2* Region effect: n.s. F(1,12)=0.062; Genotype effect: P<0.0001 F(1,12)=40.16; Region x Genotype: n.s. F(1,12)=0.022). In **(a-d)** lines connect paired samples (i.e. dOPC and vOPC samples from the same mouse). **(e,f)** Representative images of anti-γH2AX immunolabeling in the CC and dSVZ of P14 Emx1^{Cre};Cit-k$^{fl/fl}$ **(e)** and in the vSVZ and POA of P14 Nkx2.1^{Cre};Cit-k$^{fl/fl}$ **(f)**. Scale bars: 5 µm in **c**; 50 µm in **e,f** . Abbreviations: CC, corpus callosum; dOPCs, dorsal oligodendrocyte progenitor cells; dSVZ, dorsal subventricular zone; vSVZ, ventral

subventricular zone; vOPCs, ventral oligodendrocyte progenitor cells. Source data are provided as a Source Data file.

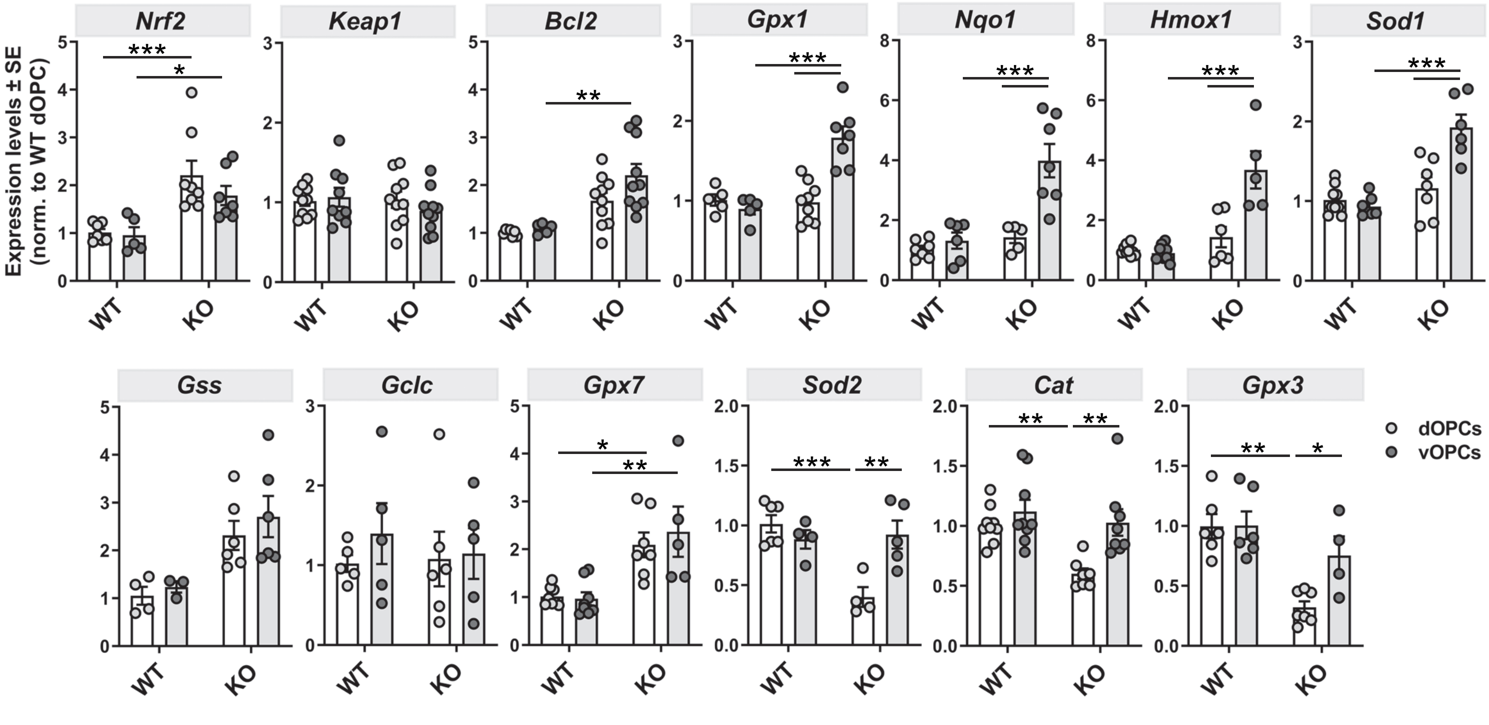

Supplementary Figure 7. qRT-PCR expression analysis of Nrf2, Keap1 and Nrf2-target genes in dorsal and ventral OPCs isolated from P10 WT and *Cit-k KO* mouse forebrain

Supplementary Figure 7. qRT-PCR expression analysis of Nrf2, Keap1 and Nrf2-target genes in dorsal and ventral OPCs isolated from P10 WT and *Cit-k KO* mouse forebrain

Dot plots referring to Figure 6**j**. Data are mean ± SE. Differences were assessed by Two-way ANOVA (see Suppl. Table 1 for P and F values of each comparison). *, $P<0.05$; **, $P<0.01$; ***, $P<0.001$. Source data are provided as a Source Data file.

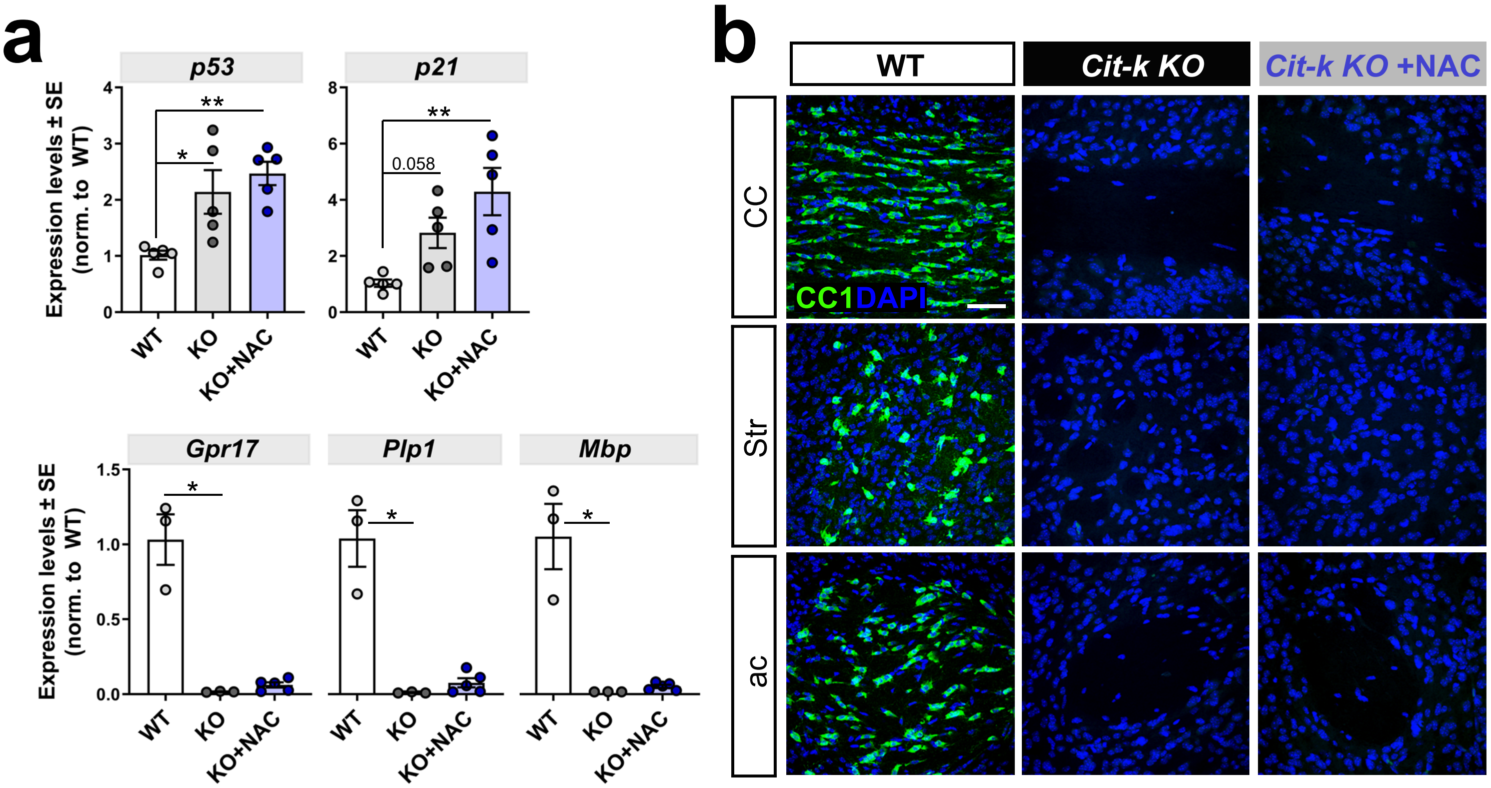

Supplementary Figure 8. Postnatal NAC treatment does not restore the expression of differentiating and mature oligodendrocyte markers in *Cit-k KO* mouse forebrain

Supplementary Figure 8. Postnatal NAC treatment does not restore the expression of differentiating and mature oligodendrocyte markers in *Cit-k KO* mouse forebrain

(a) Quantification (qRT-PCR) of the mRNAs of *p53*, *p21*, *Gpr17*, *Plp1* and *Mbp* in the ventral forebrain of P10 WT, *Cit-k KO* and NAC-treated *Cit-k KO* mice (n=3-5/group; Kruskal-Wallis test followed by Dunn's Multiple Comparison Test; *p53*, P=0.0021, $\chi^2(2)=9.500$; *p21*, P=0.0006, $\chi^2(2)=10.22$; *Gpr17*, P=0.0179, $\chi^2(2)=8.048$; *Plp1*, P=0.0006, $\chi^2(2)=8.727$; *Mbp*, P=0.0127, $\chi^2(2)=8.727$). *, P<0.05; **, P<0.01; ***, P<0.001. (b) Representative images of anti-CC1 (green) immunolabeling in P10 WT, *Cit-k KO* and NAC-treated *Cit-k KO* mouse major white matter tracts. DAPI (blue) counterstains cell nuclei. Scale bar: 5 µm. Source data are provided as a Source Data file.

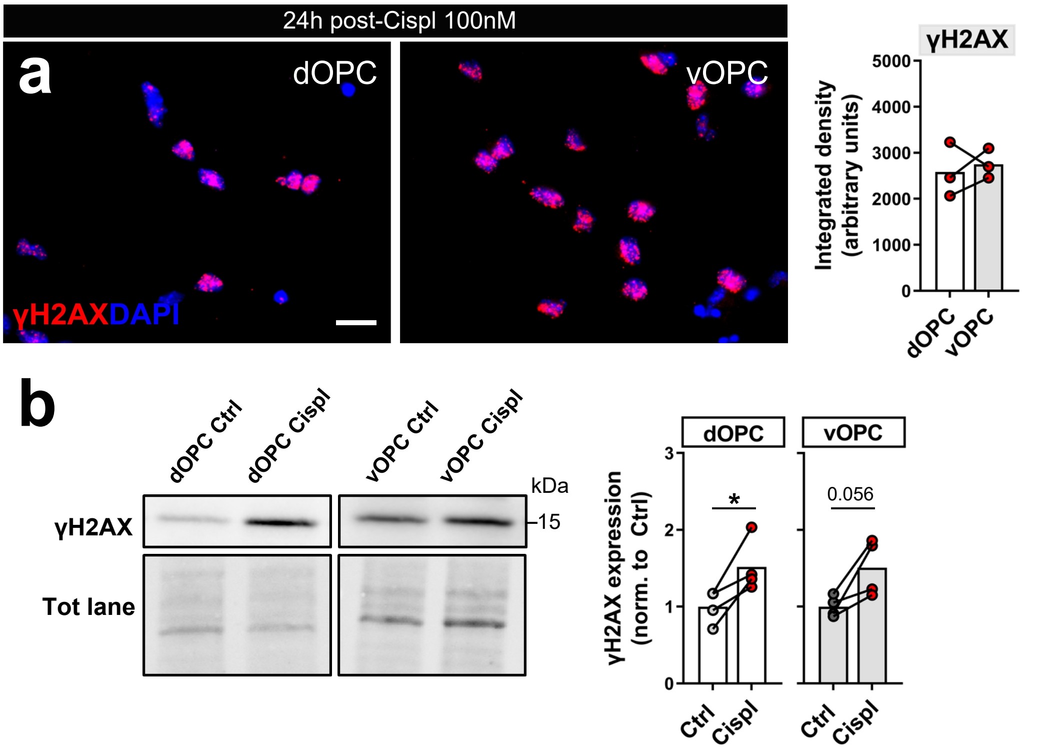

Supplementary Figure 9. DNA damage in cisplatin-treated WT mouse OPCs

Supplementary Figure 9. DNA damage in cisplatin-treated WT mouse OPCs

(a) Representative images of γH2AX staining (red) in WT mouse dOPCs and vOPCs 24 hours after incubation with cisplatin 100μM (DAPI in blue counterstains cell nuclei) and quantification of γH2AX integrated density (n=3 coverslips/each, dOPCs: 79 cells, vOPCs:158 cells; two-tailed Wilcoxon matched-pairs signed rank test, n.s.). Lines connect paired samples. Data are mean ± SEM. Scale bar: 10 μm. **(b)** Western Blot analysis of γH2AX expression in WT mouse dOPCs and vOPCs 24 hours after incubation with cisplatin 100μM compared to relative controls (vehicle). N=4 independent experiments. Two-tailed Paired t-test, dOPC: $P=0.0383$, $t(3)=3.543$; vOPC: $P=0.0567$, $t(3)=3.020$. Abbreviations: dOPCs, dorsal oligodendrocyte progenitor cells; vOPCs, ventral oligodendrocyte progenitor cells. Source data are provided as a Source Data file.

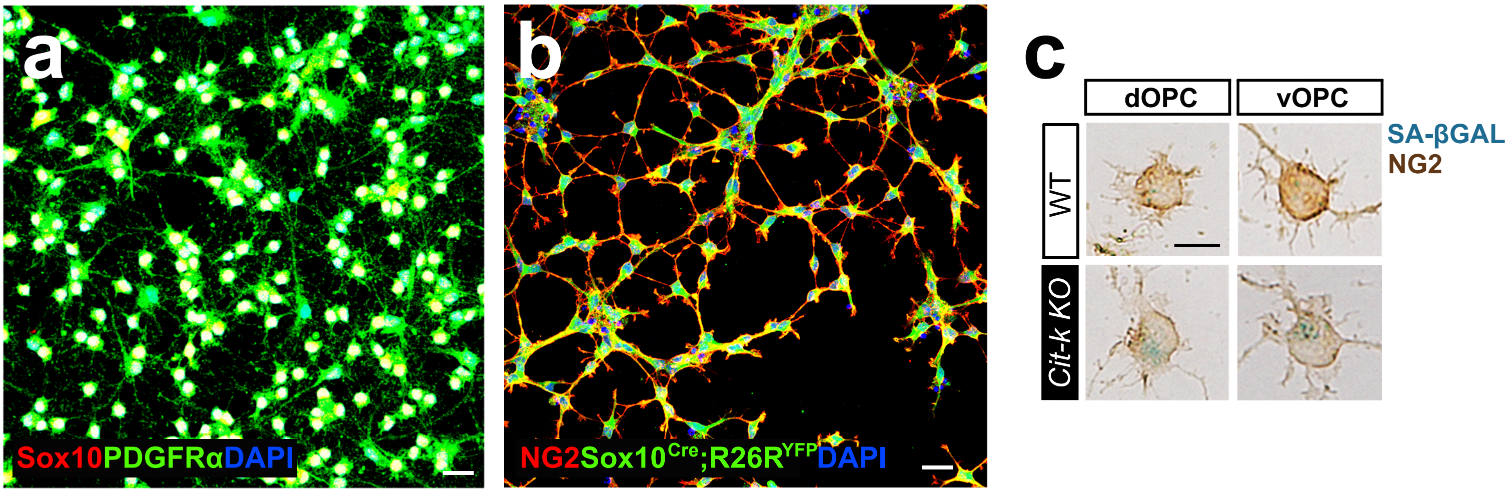

Supplementary Figure 10. Validation of the MACS isolation of OPCs

Supplementary Figure 10. Validation of the MACS isolation of OPCs

(a) Representative image of PDGFRα (green) and Sox10 (red) immunolabeling in MACSorted cells. About 98% of the MACSorted cells showed positivity for these typical oligodendroglial markers. **(b)** As a further evidence of the virtually exclusive oligodendroglia identity of the isolated cells, we MACSorted cells from $Sox10^{Cre}$;$R6R^{YFP}$ mouse forebrain. The obtained cell population was 100% YFP^{+} (green) and $NG2^{+}$(red). DAPI in blue counterstains cell nuclei. (5) Representative image of NG2-positivity (brown) of MACSorted OPCs used for SA-βGAL (blue) quantification (in Fig.4**h**). Scale bars: 10 μm in **a**,**b**; 5 μm in **c**.

Supplementary Table 1. Statistics

Figure	Applied Test	n	P value	Statistics	Post hoc analyses	Post hoc results
	ANCOVA (Analysis of Covariance)	KO dors ctx P3=3<br>KO dors ctx P14=3<br>KO Str P3=3<br>KO Str P14=3	Region effect: n.s.<br>Time effect: n.s<br>Region x Time: P=0.019	Region: F(1)=3.205<br>Time: F(1)=0.010<br>Region x Time: F(1)=9.288		
2**b**	Two-way Anova	WT P3=5<br>KO P3=5<br>WT P14=5<br>KO P14=5	Genotype effect: P<0.0001<br>Time effect: P<0.0001<br>Genotype x Time: P=0.0013.	Genotype: F(1,16)=283.4<br>Time: F(1,16)=45.59<br>Genotype x Time: F(1,16)=15.07	Bonferroni's Multiple Comparison Test	WT P3 vs KO P3: P<0.001<br>WT P14 vs KO P14: P<0.001<br>WT P3 vs WT P14: n.s.<br>KO P3 vs KO P14: P<0.001
2**c**	Two-way Anova	WT P3=5<br>KO P3=5<br>WT P14=5<br>KO P14=5	Genotype effect: P<0.0001<br>Time effect: P<0.0001<br>Genotype x Time: P<0.0001	Genotype: F(1,16)=455.0<br>Time: F(1,16)=129.9<br>Genotype x Time: F(1,16)=49.74	Bonferroni's Multiple Comparison Test	WT P3 vs KO P3: P<0.001<br>WT P14 vs KO P14: P<0.001<br>WT P3 vs WT P14: P<0.001<br>KO P3 vs KO P14: P<0.05
2**d**	Two-way Anova	WT P3=5<br>KO P3=5<br>WT P14=5<br>KO P14=5	Genotype effect: P<0.0001<br>Time effect: P=0.0096<br>Genotype x Time: n.s.	Genotype: F(1,16)=101.1<br>Time: F(1,16)=8.636<br>Genotype x Time: F(1,16)=3.872	Bonferroni's Multiple Comparison Test	WT P3 vs KO P3: P<0.001<br>WT P14 vs KO P14: P<0.001<br>WT P3 vs WT P14: P<0.01<br>KO P3 vs KO P14: n.s.
2**e**	Two-way Anova	WT P3=5<br>KO P3=5<br>WT P14=5<br>KO P14=5	Genotype effect: P<0.0001<br>Time effect: n.s.<br>Genotype x Time: n.s.	Genotype: F(1,16)=63.92<br>Time: F(1,16)=0.038<br>Genotype x Time: F(1,16)=2.113	Bonferroni's Multiple Comparison Test	WT P3 vs KO P3: P<0.001<br>WT P14 vs KO P14: P<0.001<br>WT P3 vs WT P14: n.s.<br>KO P3 vs KO P14: n.s.
3**d**	Mann Whitney U test (2-tailed)	WT=4<br>KO=3	YFP+ dSVZ cells: P=0.0571<br>YFP+ dOPCs: n.s.			
3**e**	Mann Whitney U test (2-tailed)	WT=4<br>KO=3	n.s.			
3**i**	Mann Whitney U test (2-tailed)	WT=6<br>KO=4	YFP+ dSVZ cells: P=0.0095<br>YFP+ dOPCs: n.s.			
3**j**	Mann Whitney U test (2-tailed)	WT=6<br>KO=4	P=0.0143			
3**n**	Unpaired t test (2-tailed)	WT=5<br>KO=5	YFP+: n.s.<br>YFP-: P<0.0001	YFP+: t(8)=1.732<br>YFP-: t(8)=9.878		
3**r**	Mann Whitney U test (2-tailed)	WT=3<br>KO=3	Dors. Ctx: n.s.<br>CC: n.s.<br>B-L Ctx: n.s.<br>Str: n.s.<br>POA: n.s.			

4**a**	Chi square test	**P3:** KO=4; WT=3<br>KO: Dors ctx= 561 cells;<br>CC= 276 cells;<br>Str= 361 cells;<br>POA= 204 cells<br><br>**P10:** KO=4; WT=2<br>KO: Dors ctx= 540 cells;<br>CC= 390 cells;<br>Str= 392 cells;<br>POA= 625 cells	**P3:** P=0.0156<br>(KO Dors.ctx vs KO CC: n.s.; KO Dors.ctx vs KO Str: P=0.0152; KO Dors.ctx vs KO POA: P=0.0427; KO CC vs KO Str: P=0.0137; KO CC vs KO POA:P=0.0348; KO Str vs KO POA: n.s.)<br><br>**P10:** P<0.0001<br>(KO Dors.ctx vs KO CC: n.s.; KO Dors.ctx vs KO Str: n.s.; KO Dors.ctx vs KO POA: P<0.0038; KO CC vs KO Str: P=0.0016; KO CC vs KO POA:P<0.0001; KO Str vs KO POA: n.s.)	**P3:** $\chi^2(3)=10.38$<br><br>**P10:** $\chi^2(3)=23.20$		
4**f**	Chi square test	WT dOPCs=3 (389 cells);<br>WT vOPCs=4 (510 cells);<br>KO dOPCs=4 (533 cells);<br>KO vOPCs=4<br>(476 cells)	P<0.0001<br><br>WT dOPCs vs WT vOPCs: P=0.030;<br><br>WT dOPCs vs KO dOPCs: P<0.001;<br><br>WT vOPCs vs KO vOPCs: P<0.0001;<br><br>KO dOPCs vs KO vOPCs: P<0.0001	$\chi^2(3)=456.9$<br><br>WT dOPCs vs WT vOPCs: $\chi^2(1)=4.709$<br><br>WT dOPCs vs KO dOPCs: $\chi^2(1)=11.21$<br>;<br><br>WT vOPCs vs KO vOPCs: $\chi^2(1)=337.1$<br><br>KO dOPCs vs KO vOPCs: $\chi^2(1)=186.8$		
4**k**	Paired t test (2-tailed)	WT=4<br>KO=4	p53: n.s.<br>Phospho-p53/p53: n.s.<br>p21: P=0.0249<br>p16: P=0.0041<br>p27: P=0.0449	p53: t(3)=0.947<br>Phospho-p53/p53: t(3)=1.695<br>p21: t(3)=4.185<br>p16: t(3)=8.017<br>p27: t(3)=3.325		
4**l**	Two-way Anova	WT dOPC=4-7<br>WT vOPC=4-7<br>KO dOPC=5-6<br>KO v OPC=5-6<br>(*see raw data file enclosed)	**Puma (Bbc3)**<br>Genotype: P=0.0003<br>Region: P=0.0131<br>Genotype x Region: P=0.002<br><br>**p21**<br>Genotype: P=0.0012<br>Region: P=0.0060<br>Genotype x Region: P=0.0068<br><br>**p16**<br>Genotype: P=0.0034<br>Region: n.s.<br>Genotype x Region: n.s.	**Puma (Bbc3)**<br>Genotype: F(1,16)=21.71<br>Region: F(1,16)=7.781<br>Genotype x Region: F(1,16)=13.58<br><br>**p21**<br>Genotype: F(1,19)=14.61<br>Region: F(1,19)=9.545<br>Genotype x Region: F(1,19)=9.205<br><br>**p16**<br>Genotype: F(1,15)=12.02<br>Region: F(1,15)=0.994<br>Genotype x Region: F(1,15)=1.047	Bonferroni's Multiple Comparison Test	**Puma (Bbc3)**<br>WT dOPC vs WT vOPC: n.s.<br><br>WT dOPC vs KO dOPC: P<0.001<br><br>WT vOPC vs KO vOPC: n.s.<br><br>KO dOPC vs KO vOPC: P<0.001<br><br>**p21**<br>WT dOPC vs WT vOPC: n.s.<br><br>WT dOPC vs KO dOPC: n.s.<br><br>WT vOPC vs KO vOPC: P<0.001<br><br>KO dOPC vs KO vOPC: P<0.01<br><br>**p16**<br>WT dOPC vs WT vOPC: n.s.

			**Glb1**<br>Genotype: P=0.002<br>Region: P=0.021<br>Genotype x Region: n.s.<br><br>**Ecrg4**<br>Genotype: P<0.0001<br>Region: P<0.0001<br>Genotype x Region: P<0.0001	**Glb1**<br>Genotype: F(1,18)=13.33<br>Region: F(1,18)=6.441<br>Genotype x Region: F(1,18)=3.332<br><br>**Ecrg4**<br>Genotype: F(1,17)=146.85<br>Region: F(1,17)=202.23<br>Genotype x Region: F(1,17)=126.7		WT dOPC vs KO dOPC: n.s.<br><br>WT vOPC vs KO vOPC: 0.0308.<br><br>KO dOPC vs KO vOPC: n.s.<br><br>**Glb1**<br>WT dOPC vs WT vOPC: n.s.<br><br>WT dOPC vs KO dOPC: n.s.<br><br>WT vOPC vs KO vOPC: p<0.01<br><br>KO dOPC vs KO vOPC: P<0.01<br><br>**Ecrg4**<br>WT dOPC vs WT vOPC: n.s.<br><br>WT dOPC vs KO dOPC: n.s.<br><br>WT vOPC vs KO vOPC: P<0.001<br><br>KO dOPC vs KO vOPC:P<0.001
4**m**	Chi square test	WT vOPCs=4 (1071 cells);<br>KO vOPCs=4 (804 cells)	P=0.0007	$\chi^2(1)=11.60$		
5**b**	Unpaired t test (2-tailed)	Sox10Cre=4<br>Sox10Cre;Cit-k fl/fl=5	Dors. Ctx: P=0.0073<br>CC: P=0.0038<br>Str: n.s.<br>POA: n.s.	Dors. Ctx: t(7)=3.741<br>CC: t(7)=4.249<br>Str: t(7)=1.394<br>POA: t(7)=0.251		
5**c**	Chi square test	Sox10Cre: 3<br>Sox10Cre; Cit-k fl/fl: 5<br>(about 1000 cells/region/mouse)	Dors.Ctx+CC: P<0.0001<br><br>Str+POA: n.s.	Dors. Ctx+CC: $\chi^2(1)=15.98$<br><br>Str+POA: $\chi^2(1)=0.022$		
5**e**	Paired t test (2-tailed)	WT=4<br>KO=4	Bbc3: n.s.<br>p21: P=0.049<br>Ecrg4: P=0.011	Bbc3: t(3)=2.785<br>p21-: t(3)=3.204<br>Ecrg4: t(3)=5.744		
5**h**	Mann Whitney U test (2-tailed)	Emx1Cre;R26RYFP=4<br>Emx1Cre;R26RYFP;Cit-k fl/fl= 4	tot OPCs:<br>Dors. Ctx: n.s.<br>CC: n.s.<br><br>YFP+ OPCs:<br>Dors. Ctx: P=0.0286<br>CC: P=0.0286			
5**i**	Chi square test	Emx1Cre;R26RYFP: 3<br>Emx1Cre;R26RYFP;Cit-k fl/fl: 4<br>(about 1000 cells/mouse)	P=0.0038	$\chi^2(1)=8.362$		
5**j**	Mann Whitney U test (2-tailed)	Nkx2.1Cre;R26RYFP=4<br>Nkx2.1Cre;R26RYFP;Cit-k fl/fl= 4	tot OPCs:<br>Dors. Ctx: n.s.<br>CC: n.s.<br><br>YFP+ OPCs:<br>Dors. Ctx: n.s.<br>CC: n.s.			
6**b**	Mann Whitney U	dOPCs=3	n.s.			

	test<br>(2-tailed)	(n. cells=91)<br>vOPCs=3<br>(n. cells=94)				
6**d**	Paired t test<br>(2-tailed)	dOPCs=5<br>(n. cells=106)<br>vOPCs=5<br>(n. cells=105)	P=0.0013	t(4)=8.043		
6**e**	Two-way Anova	WT dOPC=4<br>WT vOPC=4<br>KO dOPC=4<br>KO v OPC=4	Genotype: P<0.0001<br>Region: P=0.0067<br>Genotype x Region: P=0.0204.	Genotype: F(1,21)=92.83<br>Region: F(1,21)=9.045<br>Genotype x Region: F(1,21)=6.288	Bonferroni's Multiple Comparison Test	WT dOPC vs WT vOPC: n.s.<br><br>WT dOPC vs KO dOPC: P=0.0001<br><br>WT vOPC vs KO vOPC: P=0.0004<br><br>KO dOPC vs KO vOPC: P=0.0041
6**g**	Paired t test<br>(2-tailed)	dOPCs=4<br>vOPCs=4	gH2AX: n.s.<br>NRF2: P=0.0155	gH2AX: t(3)=1.116<br>NRF2: t(3)=4.984		
6**i**	Paired t test<br>(2-tailed)	dOPCs=4<br>vOPCs=4	P=0.0087	t(3)=6.129		
6**j**<br><br>Suppl. 7	Two-way Anova	WT dOPC=4-10<br>WT vOPC=4-10<br>KO dOPC=4-10<br>KO v OPC=4-10<br>(*see raw data file enclosed)	**Nrf2**<br>Genotype: P<0.001<br>Region: n.s.<br>Genotype x Region: n.s.<br><br>**Sod1**<br>Genotype: P<0.001<br>Region: P<0.01<br>Genotype x Region: P<0.001<br><br>**Sod2**<br>Genotype: P<0.01<br>Region: n.s.<br>Genotype x Region: P<0.01<br><br>**Gpx1**<br>Genotype: P<0.01<br>Region: P<0.001<br>Genotype x Region: P<0.001<br><br>**Gpx3**<br>Genotype: P<0.001<br>Region: P<0.05<br>Genotype x Region: n.s.<br><br>**Nqo1**<br>Genotype: P<0.001<br>Region: P<0.001<br>Genotype x Region: P<0.01<br><br>**Hmox1**<br>Genotype: P<0.001<br>Region: P<0.001<br>Genotype x Region: P<0.001<br><br>**Cat**<br>Genotype: P<0.01<br>Region: P<0.01<br>Genotype x Region: n.s.<br><br>**Keap1**<br>Genotype: n.s.<br>Region: n.s.	**Nrf2**<br>Genotype: F(1,24)=20.09<br>Region: F(1,24)=1.536<br>Genotype x Region: F(1,24)=0.9577<br><br>**Sod1**<br>Genotype: F(1,25)=30.13<br>Region: F(1,25)=10.74<br>Genotype x Region: F(1,25)=16.55<br><br>**Sod2**<br>Genotype: F(1,15)= 9.645<br>Region: F(1,15)=4.524<br>Genotype x Region: F(1,15)=12.49<br><br>**Gpx1**<br>Genotype: F(1,22)=10.96<br>Region: F(1,22)=16.74<br>Genotype x Region: F(1,22)= 19.02<br><br>**Gpx3**<br>Genotype: F(1,19)=20.10<br>Region: F(1,19)=4.526<br>Genotype x Region: F(1,19)=4.196<br><br>**Nqo1**<br>Genotype: F(1,21)=18.40<br>Region: F(1,21)=15.82<br>Genotype x Region: F(1,21)=10.16<br><br>**Hmox1**<br>Genotype: F(1,27)=40.30	Bonferroni's Multiple Comparison Test	**Nrf2**<br>WT dOPC vs WT vOPC: n.s.<br><br>WT dOPC vs KO dOPC: P<0.001<br><br>WT vOPC vs KO vOPC: P<0.05.<br><br>KO dOPC vs KO vOPC: n.s.<br><br>**Sod1**<br>WT dOPC vs WT vOPC: n.s.<br><br>WT dOPC vs KO dOPC: n.s.<br><br>WT vOPC vs KO vOPC: P<0.001<br><br>KO dOPC vs KO vOPC: P<0.001<br><br>**Sod2**<br>WT dOPC vs WT vOPC: n.s.<br><br>WT dOPC vs KO dOPC: P<0.001<br><br>WT vOPC vs KO vOPC: n.s.<br><br>KO dOPC vs KO vOPC: P<0.01<br><br>**Gpx1**<br>WT dOPC vs WT vOPC: n.s.<br><br>WT dOPC vs KO dOPC: n.s.<br><br>WT vOPC vs KO vOPC: P<0.001<br><br>KO dOPC vs KO vOPC: P<0.001<br><br>**Gpx3**<br>WT dOPC vs WT vOPC: n.s.<br><br>WT dOPC vs KO dOPC: P<0.001<br><br>WT vOPC vs KO vOPC: n.s.

			Genotype x Region: n.s.<br><br>**Bcl2**<br>Genotype: P<0.0001<br>Region: n.s.<br>Genotype x Region: n.s.<br><br>**Gpx7**<br>Genotype: P<0.0001<br>Region: n.s.<br>Genotype x Region: n.s.<br><br>**Gss**<br>Genotype: P=0.002<br>Region: n.s.<br>Genotype x Region: n.s.<br><br>**Gclc**<br>Genotype: n.s.<br>Region: n.s.<br>Genotype x Region: n.s.	Region: F(1,27)=17.87<br>Genotype x Region: F(1,27)=21.82<br><br>**Cat**<br>Genotype: F(1,29)=9.132<br>Region: F(1,29)=10.47<br>Genotype x Region: F(1,29)=3.634<br><br>**Keap1**<br>Genotype: F(1,35)=1.158<br>Region: F(1,35)=0.007<br>Genotype x Region: F(1,35)=1.473<br><br>**Bcl2**<br>Genotype: F(1,28)=21.55<br>Region: F(1,28)=2.66<br>Genotype x Region: F(1,28)=1.326<br><br>**Gpx7**<br>Genotype: F(1,24)=25.88<br>Region: F(1,24)=0.219<br>Genotype x Region: F(1,24)=0.439<br><br>**Gss**<br>Genotype: F(1,15)=13.90<br>Region: F(1,15)=0.618<br>Genotype x Region: F(1,15)=0.079<br><br>**Gclc**<br>Genotype: F(1,17)=0.095<br>Region: F(1,17)=0.502<br>Genotype x Region: F(1,17)=0.241		KO dOPC vs KO vOPC: P<0.05<br><br>**Nqo1**<br>WT dOPC vs WT vOPC: n.s.<br><br>WT dOPC vs KO dOPC: n.s.<br><br>WT vOPC vs KO vOPC: P<0.001<br><br>KO dOPC vs KO vOPC: P<0.001<br><br>**Hmox1**<br>WT dOPC vs WT vOPC: n.s.<br><br>WT dOPC vs KO dOPC: n.s.<br><br>WT vOPC vs KO vOPC: P<0.001<br><br>KO dOPC vs KO vOPC: P<0.001<br><br>**Cat**<br>WT dOPC vs WT vOPC: n.s.<br><br>WT dOPC vs KO dOPC: P>0.01<br><br>WT vOPC vs KO vOPC: n.s.<br><br>KO dOPC vs KO vOPC: P>0.01<br><br>**Keap1**<br>WT dOPC vs WT vOPC: n.s.<br><br>WT dOPC vs KO dOPC: n.s.<br><br>WT vOPC vs KO vOPC: n.s.<br><br>KO dOPC vs KO vOPC: n.s.<br><br>**Bcl2**<br>WT dOPC vs WT vOPC: n.s.<br><br>WT dOPC vs KO dOPC: n.s.<br><br>WT vOPC vs KO vOPC: P=0.0033<br><br>KO dOPC vs KO vOPC: n.s.<br><br>**Gpx7**<br>WT dOPC vs WT vOPC: n.s.<br><br>WT dOPC vs KO dOPC: P=0.0185<br><br>WT vOPC vs KO vOPC: P=0.0042<br><br>KO dOPC vs KO vOPC: n.s.<br><br>**Gss**<br>WT dOPC vs WT vOPC: n.s.<br><br>WT dOPC vs KO dOPC: n.s.<br><br>WT vOPC vs KO vOPC: n.s.<br><br>KO dOPC vs KO vOPC: n.s.<br><br>**Gclc**

						WT dOPC vs WT vOPC: n.s.<br><br>WT dOPC vs KO dOPC: n.s.<br><br>WT vOPC vs KO vOPC: n.s.<br><br>KO dOPC vs KO vOPC: n.s.
6**k**	Non-linear regression dose-response curve (Log(inhibitor) vs. response curves)	WT dOPC Ctrl=6<br>WT vOPC Ctrl=6<br>WT dOPC H2O2 100 μM=3<br>WT vOPC H2O2 100 μM=3<br>WT dOPC H2O2 500 μM=5<br>WT vOPC H2O2 500 μM=6<br>WT dOPC H2O2 1 mM=3<br>WT vOPC H2O2 1 mM=3<br>KO dOPC Ctrl=6<br>KO vOPC Ctrl=6<br>KO dOPC H2O2 100 μM=7<br>KO vOPC H2O2 100 μM=5<br>KO dOPC H2O2 500 μM=4<br>KO vOPC H2O2 500 μM=4<br>KO dOPC H2O2 1 mM=3<br>KO vOPC H2O2 1 mM=3	KO dOPCs:<br>LC50=38.96 μM,<br>R^2=0.885,<br>Sy.x=0.145,<br>95% confidence interval= 15.72 to 96.59;<br><br>KO vOPCs:<br>LC50= 1481 μM,<br>Sy,x=0.159,<br>R^2=0.859,<br>95% confidence interval 457.4 to 4796;<br><br>WT dOPCs:<br>LC50= 1015 μM,<br>R^2=0.895,<br>Sy.x=0.135,<br>95% confidence interval 450.5 to 2285;<br><br>WT vOPCs:<br>LC50= 2046 μM,<br>R^2=0.907,<br>Sy.x=0.120,<br>95% confidence interval 783.7 to 5344			
6**l**	One-way Anova (Repeated Measures)	5 experiments:<br><br>KO dOPC t0=5 coverslips<br>KO dOPC 2DIV Ctrl=5<br>KO dOPC 2DIV NAC=5<br>KO dOPC 2DIV DMF=5	P<0.0001	F(3)=53.86	Bonferroni's Multiple Comparison Test	KO dOPC t0 vs KO dOPC 2DIV Ctrl: P=0.0003<br><br>KO dOPC t0 vs KO dOPC 2DIV NAC: P=0.040<br><br>KO dOPC t0 vs KO dOPC 2DIV DMF: P=0.0266<br><br>KO dOPC 2DIV Ctrl vs KO dOPC 2DIV NAC: P=0.0092<br><br>KO dOPC 2DIV Ctrl vs KO dOPC 2DIV DMF: P=0.0130<br><br>KO dOPC 2DIV NAC vs KO dOPC 2DIV DMF: n.s.
7**b**	Unpaired t test (2-tailed)	dOPCs KO=5<br>dOPCs KO+NAC=6	P=0.0007	t(9)=5.006		
7**c**	Mann Whitney U test (2-tailed)	dOPCs KO=4<br>dOPCs KO+NAC=4	P=0.0571			
7**d**	Mann Whitney U test (2-tailed)	WT=4<br>KO=4	**Dors.Ctx:** P=0.0286<br>**CC:** P=0.0286			
7**f**	Chi square test	KO=4<br>KO NAC=3	**Dors. Ctx:**<br>P<0.0001<br>**CC:** P<0.0001	**Dors. Ctx:**<br>$\chi^2(1)$=19.05<br><br>**CC:** $\chi^2(1)$: 27.26		
7**g**	Chi square test	KO=4	**Dors. Ctx:** n.s.<br>**CC:** P=0.0323	**Dors. Ctx:**<br>$\chi^2(1)$=0.00253		

		KO NAC=3		**CC:** $\chi^2(1)$: 4.581		
7**j**	Mann Whitney U test (1-tailed)*	KO=3<br>KO org=3	P=0.05			
8**b**	Two-way Anova	4 experiments:<br><br>dOPC Ctrl= 10 coverslips<br>vOPC Ctrl= 10<br>dOPC cispl 20 nM= 6<br>vOPC cispl 20 nM = 6<br>dOPC cispl 100 nM= 9<br>vOPC cispl 100 nM = 10<br>dOPC cispl 200 nM= 4<br>vOPC cispl 200 nM = 4	[Cisplatin]: P<0.0001<br>OPC origin: P<0.0001<br>[Cisplatin] x OPC origin: P=0.0006	[Cisplatin]: $F(3,51)=124.2$<br>OPC origin: $F(1,51)=33.53$<br>[Cisplatin] x OPC origin: $F(3,51)=6.833$	Bonferroni's Multiple Comparison Test	dOPC ctrl vs. vOPC ctrl: P= n.s.<br><br>dOPC cispl 20 nM vs. vOPC cispl 20 nM: P= 0.0571<br><br>dOPC cispl 100 nM vs. vOPC cispl 100 nM: P<0.0001<br><br>dOPC cispl 200 nM vs. vOPC cispl 200 nM: n.s.
8**c**	Non-linear regression dose-response curve (Log(inhibitor) vs. response curves)	4 experiments:<br><br>dOPC Ctrl= 10 coverslips<br>vOPC Ctrl= 10<br>dOPC cispl 20 nM= 6<br>vOPC cispl 20 nM = 6<br>dOPC cispl 100 nM= 9<br>vOPC cispl 100 nM = 10<br>dOPC cispl 200 nM= 4<br>vOPC cispl 200 nM = 4<br>dOPC cispl 1 μM= 4<br>vOPC cispl 1 μM= 4	dOPCs:<br>LC50= 48 nM,<br>R^2= 0.9404,<br>Sy.x=0.104,<br>95% confidence interval= 31.95 to 72.12nM<br><br>vOPCs:<br>LC50=173.7 nM,<br>R^2= 0.8267,<br>Sy.x=0.163,<br>95% confidence interval= 94.03 to 320.7 nM			
8**g**	Two-way Anova	4 experiments (4 mice), 2 coverslips Ctrl + 2 coverslips Cispl /each	Treatment: P<0.0001<br>Genotype: P=0.0049<br>Genotype x Treatment: P=0.0017	Treatment: $F(1,6)=97.68$<br>Genotype: $F(1,6)=18.76$<br>Genotype x Treatment: $F(1,6)=29.10$	Bonferroni's Multiple Comparison Test	YFP+ Ctrl vs YFP+ Cispl: P<0.0001<br><br>YFP-neg Ctrl vs YFP-neg Cispl: P=0.0385
8**g inset**	Paired t test (2-tailed)	4 experiments (4 mice), 2 coverslips Ctrl + 2 coverslips Cispl /each	P=0.0037	$t(3)=8.275$		
8**i**	Paired t test (2-tailed)	4 experiments:<br><br>dOPC cispl = 4<br>vOPC cispl = 4	gHAX: n.s.<br>p21: P=0.017<br>p16: P=0.057<br>p27: P=0.021<br>NRF2: P=0.025	gHAX: $t(3)=2.481$<br>p21: $t(3)=4.868$<br>p16: $t(3)=2.975$<br>p27: $t(3)=4.449$<br>NRF2: $t(3)=4.175$		
8**j**	Paired t test (2-tailed)	5 experiments:<br><br>dOPC cispl = 5<br>vOPC cispl = 5	P=0.0363	$t(4)=3.097$		
8**l**	One-way Anova	5 experiments:<br><br>dOPC Ctrl=9 coverslips<br>dOPC cispl=10<br>dOPC cispl +NAC60μg/ml=6<br>dOPC cispl +NAC200μg/ml=6	P<0.0001	$F(3,27)=31.90$	Bonferroni's Multiple Comparison Test	dOPC Ctrl vs dOPC cispl: P<0.0001<br><br>dOPC cispl vs dOPC cispl+NAC60μg/ml: P=0.0026<br><br>dOPC Ctrl vs dOPC cispl+NAC60μg/ml:

						P=0.0015<br><br>dOPC cispl vs dOPC cispl+NAC200µg/ml: P<0.0001<br><br>dOPC Ctrl vs dOPC cispl+NAC200µg/ml: P=n.s.<br><br>dOPC cispl+NAC60µg/ml vs dOPC cispl+NAC200µg/ml: P=n.s.
Suppl. 1**a**	Mann Whitney U test (1-tailed)*	WT=3<br>KO=3	Gpr17: P=0.05<br>Plp1: P=0.05<br>Mbp: P=0.05			
Suppl. 2**g**	Mann Whitney U test (2-tailed)	WT=3<br>KO=3	Dors. Ctx: n.s.<br>LGE: n.s.<br>MGE: n.s.<br>AEP/POA: n.s.			
Suppl. 3**a**	Two-way Anova	dOPC P0=3<br>vOPC P0=3<br>dOPC P10=5<br>vOPC P10=5<br>dOPC P14=3<br>vOPC P14=3	Region effect: n.s.<br>Time effect: P=0.0003<br>Region x Time: n.s.	Region: F(1,16)=0.460<br>Time: F(2,16)=14.503<br>Region x Time: F(2,16)=0.002	Bonferroni's Multiple Comparison Test	dOPC P0 vs dOPC P10: P<0.01<br><br>vOPC P0 vs vOPC P10: P<0.01<br><br>dOPC P0 vs dOPC P14: P<0.05<br><br>vOPC P0 vs vOPC P14: P<0.05
Suppl. 3**b**	Paired t test (2-tailed)	dOPC= 6<br>vOPC= 6	n.s.	t(5)=0.516		
Suppl. 3**d**	Chi square test	N=3	P<0.0001<br><br>**Dors. Ctx vs CC:** P=0.0141<br><br>**Dors. Ctx vs Str:** n.s.<br><br>**Dors. Ctx vs POA:** P=0.0083<br><br>**CC vs Str:** n.s.<br><br>**CC vs POA:** P<0.0001<br><br>**Str vs POA:** P<0.0001	$\chi^2(3)$=45.63<br><br>**Dors. Ctx vs CC:** $\chi^2(1)$=6.025<br><br>**Dors. Ctx vs Str:** $\chi^2(1)$: 3.317<br><br>**Dors. Ctx vs POA:** $\chi^2(1)$: 6.958<br><br>**CC vs Str:** $\chi^2(1)$: 0.6601<br><br>**CC vs POA:** $\chi^2(1)$: 32.48<br><br>**Str vs POA:** $\chi^2(1)$: 27.78		
Suppl. 3**e**	Simple Linear Regression	N=3/region	P=0.1557 (the slope is not significantly different to 0)	Equation: Y = 0.1410*X + 2.067<br>R^2=0.1981<br>Sy.x=3.169		
Suppl. 4**c**	Unpaired t test (2-tailed)	6 DPT=3<br>14 DPT=3	Dors. Ctx: P=0.0003<br>Str/Thal: P=0.0064	Dors. Ctx: t(4)=12.15<br>Str/Thal: t(4)=5.22		
Suppl. 4**e**	Kruskal-Wallis Test	WT dors=3<br>KO dors=3<br>WT ventr=3<br>KO ventr=3	**Il1b:** n.s<br>**Tnfa:** n.s.<br>**Nos2:** n.s.<br>**Cxcl1:** n.s.	**Il1b:** $\chi^2(3)$=2.077<br>**Tnfa:** $\chi^2(3)$=0.2308<br>**Nos2:** $\chi^2(3)$=0.2308<br>**Cxcl1:** $\chi^2(3)$=0.8974		
Suppl. 5**i**	Two-way Anova	$Emx1^{Cre}$= 4<br>$Emx1^{Cre}$;Cit-$k^{fl/fl}$= 4	Genotype: P<0.0001<br>Region: n.s.<br>Genotype x Region:	Genotype: F(1,12) = 38.15<br>Region: F(1,12) = 0.1404		

			n.s.	Genotype x Region: F(1,12) = 1.612		
Suppl. 5**k**	Two-way Anova	$Emx1^{Cre}$= 4<br>$Emx1^{Cre}$;Cit-$k^{fl/fl}$= 4	Genotype: P<0.0001<br>Region: P<0.0001<br>Genotype x Region: P=0.0002	Genotype: F(1,12) = 52.36<br>Region: F(1,12) = 580.5<br>Genotype x Region: F(1,12) = 28.76		
Suppl. 6**a**	Mann Whitney U test (2-tailed)	dOPCs=3 (n. cells=91)<br>vOPCs=3 (n. cells=94)	n.s.			
Suppl. 6 **b**	Two-way Anova	WT dOPC=10<br>WT vOPC=5<br>KO dOPC=7<br>KO v OPC=7	Genotype: P<0.0001<br>Region: n.s.<br>Genotype x Region: n.s.	Genotype: F(1,25)=112.1<br>Region: F(1,25)=1.668<br>Genotype x Region: F(1,25)=0.648	Bonferroni's Multiple Comparison Test	WT dOPC vs WT vOPC: n.s.<br><br>WT dOPC vs KO dOPC: P<0.0001<br><br>WT vOPC vs KO vOPC: P<0.0001<br><br>KO dOPC vs KO vOPC: n.s.
Suppl. 6**c**	Paired t test (2-tailed)	Sox10Cre dOPC=1<br>Sox10Cre vOPC=1<br>Sox10Cre;Cit-k fl/fl dOPC=4<br>Sox10Cre;Cit-k fl/fl vOPC =4	Foci: Sox10Cre;Cit-k fl/fl dOPC vs Sox10Cre;Cit-k fl/fl vOPC: n.s.<br><br>Int. Density: Sox10Cre;Cit-k fl/fl dOPC vs Sox10Cre;Cit-k fl/fl vOPC: n.s.	Foci: t(3)=0.733<br><br>Int. Density: t(3)=1.946		
Suppl. 6**d**	Two-way Anova	Sox10Cre dOPC=4<br>Sox10Cre vOPC=4<br>Sox10Cre;Cit-k fl/fl dOPC=4<br>Sox10Cre;Cit-k fl/fl vOPC =4	***Cit***<br>Region effect: n.s.<br>Genotype effect: P<0.0001<br>Region x Genotype: n.s.<br><br>***Bach2***<br>Region effect: n.s.<br>Genotype effect: P<0.001<br>Region x Genotype: n.s.	***Cit***<br>Region: F(1,12)=0.146<br>Genotype: F(1,12)=58.0<br>Region x Time: F(1,12)=0.0989<br><br>***Bach2***<br>Region: F(1,12)=0.0625<br>Genotype: F(1,12)=40.16<br>Region x Time: F(1,12)=0.0215	Bonferroni's Multiple Comparison Test	***Cit***<br>Sox10Cre dOPC vs Sox10Cre;Cit-k fl/fl dOPC: P<0.001<br><br>Sox10Cre vOPC vs Sox10Cre;Cit-k fl/fl vOPC: P<0.01<br><br>Sox10Cre dOPC vs Sox10Cre vOPC: n.s.<br><br>Sox10Cre;Cit-k fl/fl dOPC vs Sox10Cre;Cit-k fl/fl vOPC: n.s.<br><br>***Bach2***<br>Sox10Cre dOPC vs Sox10Cre;Cit-k fl/fl dOPC: P<0.01<br><br>Sox10Cre vOPC vs Sox10Cre;Cit-k fl/fl vOPC: P<0.01<br><br>Sox10Cre dOPC vs Sox10Cre vOPC: n.s.<br><br>Sox10Cre;Cit-k fl/fl dOPC vs Sox10Cre;Cit-k fl/fl vOPC: n.s.
Suppl. 8**a**	Kruskal-Wallis test	WT=3-5<br>KO=3-5<br>KO NAC=5	**p53:** P=0.0021<br>**p21:** P=0.0006<br>**Gpr17:** P=0.0179<br>**Plp1:** P=0.0006<br>**Mbp:** P=0.0127	**p53:** $\chi^2(2)$= 9.500<br>**p21:** $\chi^2(2)$= 10.22<br>**Gpr17:** $\chi^2(2)$= 8.048<br>**Plp1:** $\chi^2(2)$= 8.727<br>**Mbp:** $\chi^2(2)$= 8.727	Dunn's Multiple Comparison Test	**p53:**<br>WT vs KO: P=0.04<br>KO vs KO NAC: n.s.<br>WT vs KO NAC: P=0.014<br><br>**p21:**<br>WT vs KO: P=0.058<br>KO vs KO NAC: n.s.

						WT vs KO NAC: P=0.0056<br><br>**Gpr17:**<br>WT vs KO: P<0.05<br>KO vs KO NAC: n.s.<br>WT vs KO NAC: n.s.<br><br>**Plp1:**<br>WT vs KO: P<0.01<br>KO vs KO NAC: n.s.<br>WT vs KO NAC: n.s.<br><br>**Mbp:**<br>WT vs KO: P<0.01<br>KO vs KO NAC: n.s.<br>WT vs KO NAC: n.s.
Suppl. 9**a**	Wilcoxon matched-pairs signed rank test (2-tailed)	1 experiment:<br>dOPCs=3 coversplis (79 cells)<br>vOPCs=3 (158 cells)	n.s.			
Suppl. 9**b**	Paired t test (2-tailed)	dOPC Ctrl=4<br>dOPC Cispl=4<br>vOPC Ctrl=4<br>vOPC Cispl=4	dOPC: P=0.0383<br>vOPC: P=0.0567	dOPC: t(3)=3.543<br>vOPC: t(3)=3.020		

* Since Gpr17 and Mbp mRNAs, and MBP protein are absent in *Cit-k KO* mouse brain, we opted for one-tailed tests to assess whether levels in WT or *KO* NAC+organotypic samples are higher than in the *KO* (they cannot be lower than 0).

Supplementary Table 2. List of the predeveloped Taqman assays or combinations of primers+UPL probes used in the present study

Gene	*Taqman assay (Applied Biosystems)*	*Primers+UPL probe (Roche Diagnostics)*
**BTB and CNC homology 2 (Bach2)**	Mm00464379_m1	
**β-actin (β-Act)**	Mm00607939_s1	
**Beta-galactosidase 1 (Glb1)**		FW: cacctttttacgtgggcaac<br>RV: gccattgatccatacttgacc<br>Probe #58
**Catalase (Cat)**		FW: ccttcaagttggttaatgcaga<br>RV: caagtttttgatgccctggt<br>Probe #34
**Cdkn1a (p21)**	Mm00432448_m1	
**Cdkn2a (p16)**	Mm0494449_m1	
**Cit (exon1)**	Mm00801509_m1	
**Cit (exon2-3)**		FW: aacccgccctgatgaagatg<br>RV: ccacaaggcttcgaacttcg<br>Probe #24
**Chemokine (C-X-C motif) ligand 1 (Cxcl1)**	Mm00436454_m1	
**Esophageal Cancer-Related Gene 4 (Ecrg4)**		FW: catgagcacctcgtctgc<br>RV: ttgtttccacttatgccatctg<br>Probe #21
**G-coupled receptor 17 (Gpr17)**	Mm02619401_s1	
**Glutamate-cysteine ligase catalytic subunit (Gclc)**		FW: agatgatagaacacgggaggag<br>RV: tgatcctaaagcgattgttcttc<br>Probe #1
**Glutathione peroxidase 1 (Gpx1)**		FW: tttcccgtgcaatcagttc<br>RV: tcggacgtacttgagggaat<br>Probe #2
**Glutathione peroxidase 3 (Gpx3)**		FW: gtgaacggggagaaagagc<br>RV: tgagcccaggagttctgc<br>Probe #51
**Glutathione peroxidase 7 (Gpx7)**		FW: caccctgccttcaagtaccta<br>RV: tttccgtctgggtccacta<br>Probe: #12
**Glutathione synthetase (Gss)**		FW: ctcggagctgggtatttttg<br>RV: gctttggttcgaagcaggt<br>Probe: #9
**Heme oxygenase 1 (Hmox1)**		FW: aggctaagaccgccttcct<br>RV: tgtgttcctctgtcagcatca<br>Probe #17
**Interleukin 1β (Il1β)**	Mm00434228_m1	
**Kelch-like ECH-associated protein 1(Keap1)**		FW: ctgcactgaactgcaccag<br>RV: ggcagtgtgacaggttgaag<br>Probe #10
**Myelin basic protein (Mbp)**	Mm01262035_m1	
**Nitric Oxide Synthase 2 (Nos2)**	Mm00440502_m1	
**NAD(P)H Quinone Dehydrogenase 1(Nqo1)**		FW: agcgttcggtattacgatcc<br>RV: agtacaatcagggctcttctcg<br>Probe #50
**Nuclear factor (erythroid-derived 2)-like 2 (Nrf2)**		FW: catgatggacttggagttgc<br>RV: cctccaaaggatgtcaatcaa<br>Probe #3
**p53**		FW: gcaactatggcttccacctg<br>RV: ttattgaggggaggagagtacg

		Probe #4
**p53-upregulated modulator of apoptosis (Puma/Bbc3)**	Mm00519268_m1	
**Proteolipid protein 1 (Plp1)**	Mm01297210_m1	
**Superoxide dismutase 1 (Sod1)**		FW: caggacctcattttaatcctcac<br>RV: tgcccaggtctccaacat<br>Probe #49
**Superoxide dismutase 2 (Sod2)**		FW: tgctctaatcaggacccattg<br>RV: gtagtaagcgtgctcccacac<br>Probe #3
**Tumor Necrosis Factor α (Tnfα)**	Mm00443260_g1	
