# Supplementary material for: Molecular and functional heterogeneity in dorsal and ventral oligodendrocyte progenitor cells of the mouse forebrain in response to DNA damage
Source: Nat Commun. 2022 Apr 28;13:2331. doi: 10.1038/s41467-022-30010-6 (PMC9051058; doi:10.1038/s41467-022-30010-6)
Supplement: Supplementary file 3 — Reporting Summary [file 41467_2022_30010_MOESM3_ESM.pdf]

nature portfolio

Corresponding author(s): Enrica Boda

Last updated by author(s): Mar 13, 2022

Reporting Summary

Nature Portfolio wishes to improve the reproducibility of the work that we publish. This form provides structure for consistency and transparency in reporting. For further information on Nature Portfolio policies, see our Editorial Policies and the Editorial Policy Checklist.

Statistics

For all statistical analyses, confirm that the following items are present in the figure legend, table legend, main text, or Methods section.

n/a	Confirmed	
☐	☒	The exact sample size (*n*) for each experimental group/condition, given as a discrete number and unit of measurement
☐	☒	A statement on whether measurements were taken from distinct samples or whether the same sample was measured repeatedly
☐	☒	The statistical test(s) used AND whether they are one- or two-sided<br>*Only common tests should be described solely by name; describe more complex techniques in the Methods section.*
☐	☒	A description of all covariates tested
☐	☒	A description of any assumptions or corrections, such as tests of normality and adjustment for multiple comparisons
☐	☒	A full description of the statistical parameters including central tendency (e.g. means) or other basic estimates (e.g. regression coefficient) AND variation (e.g. standard deviation) or associated estimates of uncertainty (e.g. confidence intervals)
☐	☒	For null hypothesis testing, the test statistic (e.g. *F*, *t*, *r*) with confidence intervals, effect sizes, degrees of freedom and *P* value noted<br>*Give P values as exact values whenever suitable.*
☒	☐	For Bayesian analysis, information on the choice of priors and Markov chain Monte Carlo settings
☒	☐	For hierarchical and complex designs, identification of the appropriate level for tests and full reporting of outcomes
☒	☐	Estimates of effect sizes (e.g. Cohen's *d*, Pearson's *r*), indicating how they were calculated

Our web collection on statistics for biologists contains articles on many of the points above.

Software and code

Policy information about availability of computer code

Data collection	LAS AF 4.0 (Leica Microsystems, Wetzlar, Germany) and EZ-C1 Ver3.90 (Nikon, Melville, NY) softwares were used for confocal imaging. Zeiss ZEN software was used to acquire images with the Axio Scan Z.1 microscope slide scanner (Zeiss, Oberkochen, Germany). Neurolucida (MicroBrightfield, Colchester, VT) software was used for acquisition of fluorescence imaging. Soft-Imaging-System (SIS, Münster, Germany) was used to acquire electron microscopy images. Real Time data were collected on The Applied Biosystems StepOnePlus Real-Time PCR System with StepOne™ Software. Western blots were imaged using Image Lab software 3.0 on a ChemiDocTM (Bio-Rad).
Data analysis	Quantitative evaluations on confocal or fluorescence microscopy images were performed with Neurolucida (MicroBrightfield, Colchester, VT) or ImageJ (Research Service Branch, National Institutes of Health, Bethesda, MD; available at http://rsb.info.nih.gov/ij/) softwares. Statistical analyses were carried out with GraphPad Prism 9 (GraphPad software, Inc). Western blots were analyzed using Image Lab software (Bio-Rad). Real Time data were analyzed by using Microsoft Excel (Microsoft Office 365). Adobe Photoshop 6.0 (Adobe Systems, San Jose, CA) was used to assemble the final plates.

For manuscripts utilizing custom algorithms or software that are central to the research but not yet described in published literature, software must be made available to editors and reviewers. We strongly encourage code deposition in a community repository (e.g. GitHub). See the Nature Portfolio guidelines for submitting code & software for further information.

Data

Policy information about availability of data

All manuscripts must include a data availability statement. This statement should provide the following information, where applicable:

- Accession codes, unique identifiers, or web links for publicly available datasets
- A description of any restrictions on data availability
- For clinical datasets or third party data, please ensure that the statement adheres to our policy

Figures that have associated raw data: Figure 2b,c,d,e; Figure 3d,e,i,j,n,r; Figure 4a,i,k,l,m; Figure 5b,c,e,h,i,l; Figure 6b,d,e,g,i,j,k,m; Figure 7b,c,d,f,g,j; Figure 8b,c,g,i,j,l; Suppl. Figure 1a; Suppl. Figure 2g; Suppl. Figure 3a,b,d,e; Suppl. Figure 4c,e; Suppl. Figure 5i,k; Suppl. Figure 6a,b,c,d; Suppl. Figure 7; Suppl. Figure 8a; Suppl. Figure 9a,b

Field-specific reporting

Please select the one below that is the best fit for your research. If you are not sure, read the appropriate sections before making your selection.

☒ Life sciences ☐ Behavioural & social sciences ☐ Ecological, evolutionary & environmental sciences

For a reference copy of the document with all sections, see nature.com/documents/nr-reporting-summary-flat.pdf

Life sciences study design

All studies must disclose on these points even when the disclosure is negative.

Sample size	Individual data are represented in all histogram/graph. In all histological quantifications, at least 3 animals and at least 3 sections per animal were analyzed for each time point or experimental condition. For gene expression analyses, 4-10 samples/genotype or condition were used. For functional experiments with H2O2, 4-6 samples/group was used. For in vitro analyses with cisplatin, 4-10 samples/group were used. See Supplementary table 1 for the exact sample size (and n of analyzed cells) of each figure. The sample sizes for tissue analyses were determined based on the practice in the field (doi: 10.1038/s41467-021-22532-2; doi: 10.1002/glia.22750; doi: 10.1038/s41598-021-86673-6), the variability obtained from pilot studies, and with the aim to reduce redundancy (3R rule according to IACUC regulations) while providing sufficient power to detect the differences between control and experimental groups. The sample sizes for slice and dissociated culture analyses were determined based on the practice in the field (doi: 10.1038/s41467-021-22532-2; doi: 10.1038/s41598-021-86673-6), the variability obtained from pilot studies.
Data exclusions	For lineage tracing in NG2CreERTM; R26RYFP mice, YFP+ cells with pericyte morphology (that sporadically appeared together with OPCs, since NG2 promoter is active in both cell types at embryonic ages) were excluded from counts. This was a pre-established criterion prior to counting, so no data were excluded from the manuscript.
Replication	Each experiment and histological analysis was repeated at least three times independently with similar results. For tissue analyses, mice were obtained from more than two different litters. Representative immuno-/histochemical staining images are representative of at least 3 sections from at least 3 different animals/group. For in vitro studies, data represents 3-5 biological repeats, as detailed in Supplementary Table 1.
Randomization	Mice for in vivo studies, isolated OPC samples and ex vivo slices were randomly assigned to experimental time points/conditions.
Blinding	Since the gross neuroanatomy of Cit-k KO and Emx1Cre;Cit-k fl/fl mice macroscopically differs from that of WT mice, data collection in these mouse lines could not be performed blind. Yet, data collection were performed to eliminate bias by randomly capturing image fields based on DAPI channel and then opening the other channels for quantification. In all the other analyses the experimenter was blind to the genotype of the samples.

Reporting for specific materials, systems and methods

We require information from authors about some types of materials, experimental systems and methods used in many studies. Here, indicate whether each material, system or method listed is relevant to your study. If you are not sure if a list item applies to your research, read the appropriate section before selecting a response.

Materials & experimental systems

n/a	Involved in the study	
☐	☒	Antibodies
☒	☐	Eukaryotic cell lines
☒	☐	Palaeontology and archaeology
☐	☒	Animals and other organisms
☐	☒	Human research participants
☒	☐	Clinical data
☒	☐	Dual use research of concern

Methods

n/a	Involved in the study	
☒	☐	ChIP-seq
☒	☐	Flow cytometry
☒	☐	MRI-based neuroimaging

Antibodies

Antibodies used

Primary antibodies used for analyses on mouse and human slices/cells: Rb Olig2 (1:500, Millipore, Billerica, MS, USA, AB9610); Rb NG2 (1:200, Millipore, Billerica, MS, USA, AB5320); Rat PDGFRα (APA-5 clone, 1:300, BD Biosciences, San Jose, CA, USA, 55874); Mouse MBP (Smi-99 clone, 1:1000 Sternberger ,808401); Chicken GFP (1:700, AvesLabs Inc. Davis CA, USA, GFP-1020); Mouse cleaved-caspase 3 (1:150, Cell Signaling Technology, Danvers, MA, USA, D9661S); Rb GFAP (1:1000, Dakopatts, Agilent, Santa Clara, CA, Z334); Rb GPR17 affinity-purified antibody (1:100; produced by Dr. Patrizia La Rosa, CNR Institute of Neuroscience, Milan, Italy); Rb γH2AX (Ser139 20E3, 1:100; Cell Signaling Technology, Danvers, MA, USA, 97185); Rb NRF2 (1:200; Abcam, Cambridge, GB, Ab31163); Rat AN2 (rat homologue of NG2, 1:100; kind gift of Miltenyi Biotech GmbH, Bergisch Gladbach, DE, and Prof. J. Trotter, Johannes Gutemberg University of Mainz, DE); Rb Sox10 (1:1000, Sigma Aldrich, HPA068898), rat BrdU (1:500; Abcam, Cambridge, UK,AB6326); Rb PH3 (1:500, Millipore, Burlington, MA, USA, 06-570); mouse CC1 (1:1500, Millipore, Burlington, MA, USA, OP80); Rb BLBP (1:200, Millipore, Billerica, MS, USA; ABN14).

For Western Blot: Mouse anti-CIT (1:1000, Transduction Laboratories, BD Biosciences, San Jose, CA, USA, 611377); mouse anti-MBP (1:1000, Millipore, Billerica, MS, USA, MAB382), rabbit anti-p21 (1:1000, Santa Cruz – MW: 21 kDa; sc-6246), rabbit Phospho-p53 (1:1000, Cell Signaling Technology, Danvers, MS, USA – MW: 53 KDa; 9288), rabbit γH2AX (1:1000, Cell Signaling Technology, Danvers, MS, USA – MW: 15 KDa; 9718), rabbit NRF2 (1:500, Cell Signaling Technology, Danvers, MS, USA – MW: 120 KDa; AB31163), rabbit p16 (1:1000, Abcam – MW: 16 kDa; AB211542), mouse anti-p53 (1:1000, Cell Signaling Technology, Danvers, MS, USA – MW: 53 kDa; 9282), mouse p27 (1:1000, BD Biosciences, San Jose, CA, USA – MW: 27kDa; G173-524), mouse anti-αTubulin (1:5000, Sigma-Aldrich, Saint Louis, MS, USA, T5168), mouse anti-βActin (1:5000, Sigma-Aldrich, Saint Louis, MS, USA, A2228), rabbit anti-glyceraldehyde 3- phosphate dehydrogenase (GAPDH, 1:1000, Cell Signaling Technology, Danvers, MS, USA – MW: 37 kDa; 97166).
For MACS: Rat anti-PDGFRa conjugated microbeads (Miltenyi Biotec GmbH, Bergisch Gladbach, DE, 130-101-502).

Secondary antibodies: Alexafluor488-conjugated anti-Chicken (IgG, goat, Molecular Probes Life Technologies A11039, 1:500), anti-Mouse (IgG, donkey, Molecular Probes Life Technologies A21202, 1:500), anti-Rabbit (IgG, donkey,Molecular Probes Life Technologies A21206, 1:500), anti-Rat (IgG, donkey, Jackson ImmunoResearch Laboratories, West Grove, PA 712-545-153, 1:500); Cy3-conjugated anti-Mouse (IgG, donkey, Jackson ImmunoResearch Laboratories, 715-167-003 1:100) and anti-Rat (IgG, donkey, Jackson ImmunoResearch Laboratories, 715-165-153, 1:500); Alexafluor555-conjugated anti-Rb (IgG, goat, Molecular Probes Life Technologies A31572, 1:500).

Validation

Antibodies against NG2, AN2, PDGFRα, MBP,GFAP, Olig2, GPR17, GFP, CC1, Sox10, BrdU, PH3 and all secondary antibodies listed above were previously validated in Boda et al 2011 Glia; Boda et al., 2015 Glia; Lorenzati et al., 2021 Sci Rep. Antibodies against γH2AX, cleaved-caspase 3, CIT, p53, phospho-p53, p21, p16, p27, αTubulin, βActin, GAPDH were previously validated in Bianchi et al., 2017 Cell Reports and Pallavicini et al., 2018 Cancer Res. Rat anti-PDGFRa conjugated microbeads used for MACSorting were tested and validated in Boda et al., 2015 Glia.
All other antibodies have been validated as described in the the specification sheets on the providers' websites:
BLBP: https://www.merckmillipore.com/IT/it/product/Anti-Brain-lipid-binding-protein-Antibody,MM_NF-ABN14?ReferrerURL=https%3A%2F%2Fwww.bing.com%2F&bd=1
NRF2: https://www.abcam.com/nrf2-antibody-ab31163.html

Animals and other organisms

Policy information about studies involving animals; ARRIVE guidelines recommended for reporting animal research

Laboratory animals

Groups of 4–5 mice were housed in transparent polycarbonate cages (Tecnoplast, Buggirate, Italy) provided with sawdust bedding, boxes/tunnels hideout as environmental enrichment and striped paper as nesting material. Food and water were provided ad libitum; environmental conditions were 12 h/12 h light/dark cycle, room temperature 21 °C±1 °C and room humidity 55%±5%. For histological and molecular analyses, OPC Magnetic-Activated Cell Sorting (MACS) and in vitro functional assays, pharmacological treatments and intracerebral injection of retroviral particles or Tat-Cre, we employed germinal Cit-k KO and age-matched wild-type (WT) mice. For lineage tracing analyses in Cit-k KO mice, we crossed Cit-k KO mice with either R26RYFP, Emx1Cre;R26RYFP or NG2-CreERTM;R26RYFP mouse mutants. For transplantation experiments, cells were obtained from β-actin-green fluorescent protein (GFP) mice. Emx1Cre;R26RYFP and C57BL/6J mice were used to study OPC response to cisplatin in vitro. Cit-kfl/fl mice (originally obtained from UC Davis KOMP repository as Cittm1a(KOMP)Wtsi) were crossed with Sox10Cre (B6;CBA-Tg(Sox10-cre)1Wdr/J), Emx1Cre;R26RYFP or Nkx2.1Cre;R26RYFP (C57BL/6J-Tg(Nkx2-1-cre)2Sand/J) mouse lines. Source and references for each mouse line are included in the method section. Analyses and experiments involved P0-P14 mice of both sexes, as detailed in the Methods. MACSorted OPCs were derived from P8-P10 mouse pups. Organotypic cultures were derived from P10 Cit-k KO mice. ARRIVE guidelines have been followed for reporting and included in the manuscript, such as ethical permissions, animal strains used, surgical protocols, methods of termination, all commercial providers for reagents, exact n numbers used, statistical information and p values, and relevance to human disease.

Wild animals

This study did not involve the use of any wild animals.

Field-collected samples

This study did not involve field-collected samples.

Ethics oversight

The experimental plan was designed according to the guidelines of the NIH, the European Communities Council (2010/63/EU) and the Italian Law for Care and Use of Experimental Animals (DL26/2014). It was also approved by the Italian Ministry of Health (authorization 1112/2016-PR to AB and authorization 510/2020-PR to EB) and by the Bioethical Committee of the University of Turin.

Note that full information on the approval of the study protocol must also be provided in the manuscript.

Human research participants

Policy information about studies involving human research participants

Population characteristics	This study used post-mortem tissue of human brain from a non-neurological control and a male newborn (died 1 day after birth) carrying a biallelic truncating variant of CIT-K (CIT-K fs/fs), whose pedigree, genetics and clinical data are described in Harding et al., 2016 The American Journal of Human Genetics (proband B).
Recruitment	CIT-K fs/fs individual belonged to a family from the United Arab Emirates, with two affected children who present with severe microcephaly. Genetic diagnosis showed that this subject carried a biallelic truncating variant of CIT-K, resulting in the loss of function of CIT-K. For this study, the patient was recruited without bias, based on availability and parents' willingness to participate in scientific research studies.
Ethics oversight	Parents provided written informed consent for post mortem studies that followed the approved guidelines of institutional review boards at Great Ormond St. Hospital for Children, London UK, and Children’s Hospital of Philadelphia USA.

Note that full information on the approval of the study protocol must also be provided in the manuscript.
